# The Role of Tryptophan in π Interactions in Proteins: An Experimental Approach

**DOI:** 10.1021/jacs.2c04986

**Published:** 2022-07-22

**Authors:** Jinfeng Shao, Bastiaan P. Kuiper, Andy-Mark W. H. Thunnissen, Robbert H. Cool, Liang Zhou, Chenxi Huang, Bauke W. Dijkstra, Jaap Broos

**Affiliations:** † Groningen Biomolecular Science and Biotechnology Institute (GBB), University of Groningen, Nijenborgh 7, 9747 AG Groningen, The Netherlands; ‡ Department of Chemical and Pharmaceutical Biology, University of Groningen, Antonius Deusinglaan 1, 9713 AV Groningen, The Netherlands

## Abstract

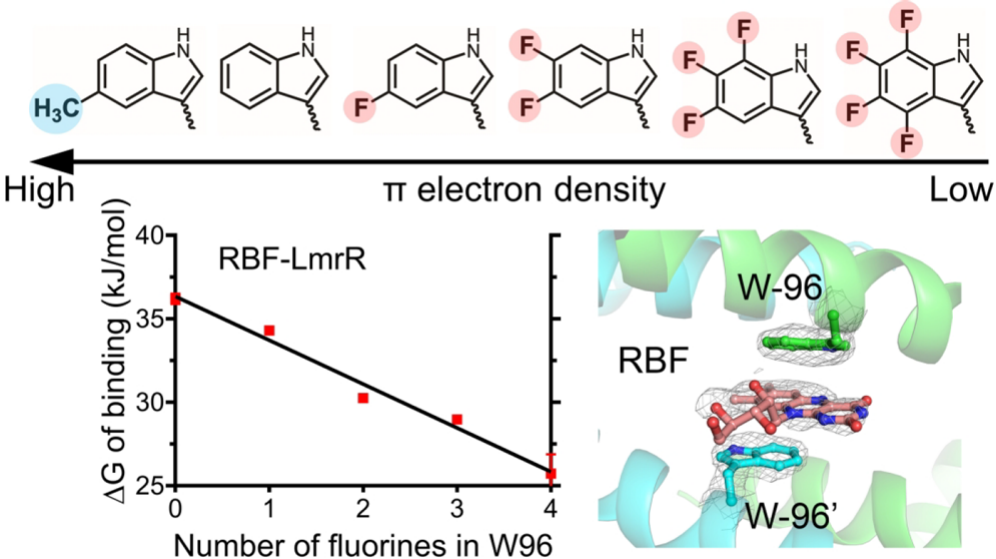

In proteins, the amino acids Phe, Tyr, and especially Trp are frequently involved in π interactions such as π–π, cation−π, and CH−π bonds. These interactions are often crucial for protein structure and protein–ligand binding. A powerful means to study these interactions is progressive fluorination of these aromatic residues to modulate the electrostatic component of the interaction. However, to date no protein expression platform is available to produce milligram amounts of proteins labeled with such fluorinated amino acids. Here, we present a *Lactococcus lactis* Trp auxotroph-based expression system for efficient incorporation (≥95%) of mono-, di-, tri-, and tetrafluorinated, as well as a methylated Trp analog. As a model protein we have chosen LmrR, a dimeric multidrug transcriptional repressor protein from *L. lactis.* LmrR binds aromatic drugs, like daunomycin and riboflavin, between Trp96 and Trp96′ in the dimer interface. Progressive fluorination of Trp96 decreased the affinity for the drugs 6- to 70-fold, clearly establishing the importance of electrostatic π–π interactions for drug binding. Presteady state kinetic data of the LmrR–drug interaction support the enthalpic nature of the interaction, while high resolution crystal structures of the labeled protein–drug complexes provide for the first time a structural view of the progressive fluorination approach. The *L. lactis* expression system was also used to study the role of Trp68 in the binding of riboflavin by the membrane-bound riboflavin transport protein RibU from *L. lactis*. Progressive fluorination of Trp68 revealed a strong electrostatic component that contributed 15–20% to the total riboflavin-RibU binding energy.

## Introduction

In proteins, aromatic amino acid side chains are often engaged in noncovalent π interactions such as π–π, cation−π, and CH−π interactions contributing to protein stability, protein–ligand interactions, catalysis, and self-assembly.^1−8^ Identifying these interactions and quantifying their strength are not straightforward. The results of mutation studies aimed at probing the interaction are difficult to interpret because of the perturbation of the local protein structure if one of the aromatic residues is replaced by a nonaromatic amino acid. A more direct experimental approach to evaluate π interactions is the progressive fluorination of aromatic amino acids originally developed for quantitating cation−π interactions in neurotransmitter-gated ion channels.^3,4,9^ In this approach an aromatic residue in the cation binding site is replaced by an analog containing one to four fluorine substituents in its aromatic side chain.^10^ Introduction of a strongly electronegative fluorine atom decreases the electron density in the aromatic π-electron cloud, thereby reducing the electrostatic attraction between the cation and the π-electron system. The electrostatic potential of the aromatic ring becomes essentially zero when four fluorine atoms are introduced in a Trp side chain or three fluorine atoms in a Phe side chain.^7^ When the labeled aromatic residue is involved in a cation−π interaction, a plot of the interaction energy against the number of fluorine atoms in the aromatic amino acid or against the in silico calculated cation−π interaction energy is expected to yield a linear free energy relationship.^9^ Linear free energy relationship analysis has become the benchmark to identify and quantitate the electrostatic component of cation−π interactions. Fluorine-labeled proteins have been successfully expressed in *Xenopus laevis* oocytes, using a site-specific, noncanonical amino acid expression system based on a chemical tRNA aminoacylation strategy.^9^ This approach allowed evaluation of cation−π interactions in many ion channels and neuroreceptors^11,12^ as well as Phe-Phe stacking in the D2 dopamine receptor.^13^ Because of the very low protein yield (pmol), only labeled proteins can be investigated of which the function can be probed by changes in the membrane current of oocytes. As an alternative of the oocyte expression system, a higher yield method for introducing fluorinated amino acids, solid-state peptide synthesis, has been used, which was successful for a few small proteins and peptides (<40 residues).^14−17^ Here, we present an expression system for the efficient biosynthetic incorporation of fluorinated Trp analogs, yielding milligram amounts of labeled proteins, using a Trp auxotroph of *Lactococcus lactis* that constitutively overexpresses the tryptophanyl tRNA synthethase of *L. lactis*. With this expression system, the role of π–π interactions in the interaction of aromatic drugs with the *L. lactis* protein LmrR was investigated in detail showing that π–π interactions play a major role in drug binding by this protein.

LmrR is a transcriptional repressor regulating the expression of the multidrug ABC transporter LmrCD.^18^ In the apo state, LmrR shows high affinity for the promotor region of LmrRCD. This affinity is reduced upon binding of a drug at its drug-binding site, enabling expression of the multidrug transporter.^18−20^ LmrR is a homodimeric protein with the drug binding site at the dimer interface (Figure S1).^19^ Central in this binding site are two Trp residues, W96 and W96′ (residue 96 from the other monomer), oriented face-to-face at 7 Å from each other. This space is sufficient for an aromatic moiety of a drug to stack between these two Trp side chains.^19^ Indeed, inspection of the LmrR-Hoechst33342, LmrR-daunomycin, and LmrR-riboflavin crystal structures suggests that π–π interactions are important for binding the drugs.^19,20^ Mutating W96 to Ala or Tyr abolishes the binding.^19^ In contrast, the importance of the π–π interactions for drug binding by LmrR has recently been challenged by Takeuchi et al.^21^ By probing the conformational dynamics of LmrR with a variety of biophysical techniques, it was concluded that drug binding by LmrR is entropy driven since an increase of conformational flexibility was observed in LmrR upon drug binding. Desolvation of the hydrophobic compound-binding pore of LmrR was suggested as another source generating entropy. Isothermal titration calorimetry data supported this conclusion by showing that the changes in enthalpy upon drug binding were small or unfavorable.

To investigate the energetics of drug binding by LmrR in more detail, and to quantify the contribution of π–π interactions to the overall binding energy, we applied the progressive fluorination approach to W96 in LmrR. Our data indicate that π–π stacking enhances the affinity of LmrR affinity for aromatic drugs by up to 70-fold. Presteady state kinetics confirm the enthalpic nature of this interaction as progressive fluorination of W96 decreases the affinity of the protein for the drug, as evidenced by an increased *k*_off_ rate of the LmrR–drug complex. Furthermore, crystal structures of labeled LmrR proteins in complex with daunomycin (Dau) showed that progressive fluorination did not significantly affect the protein conformation. A further successful application of our method was the progressive fluorination of a Trp residue in the S component of the riboflavin (RBF; vitamin B2) transport protein RibU from *L. lactis*. This allowed for the first time quantification of the electrostatic contribution of an aromatic residue in a key step of a membrane protein transport process.

## Results

### Biosynthetic Incorporation of Trp Analogs into the LmrR Protein

The LmrR protein contains two unique Trp residues, W96 in the drug binding site and W67 at a solvent exposed position, approximately 30 Å away from W96 and the drug binding site (Figure S1). Thus, W67 is not involved in drug binding. LmrR proteins, each labeled with a different Trp analog, were expressed using the *L. lactis* Trp auxotroph strain PA1002. This Trp auxotroph strain also contains the plasmid pMG36e-TrpRS, which gives constitutive overexpression of lacTrpRS. lacTrpRS is the tryptophanyl-tRNA synthetase from *L. lactis*, which features a relaxed substrate specificity for Trp analogs.^22^ Overexpression of lacTrpRS is a prerequisite for a high incorporation efficiency of the Trp analogs used in this study, except for 5-fluorotryptophan (5FW).^22^

The final expression levels of LmrR labeled with 5FW and 5,6-difluorotryptophan (5,6diFW) were similar to that of wild type protein, about 5–9 mg/L, while LmrR labeled with 5,6,7-trifluorotryptophan (5,6,7triFW), 4,5,6,7-tetrafluorotryptophan (4,5,6,7tetraFW), and 5-methyltryptophan (5MeW) gave slightly lower expression levels. The incorporation efficiency of the Trp analog was determined by MALDI-TOF after tryptic digestion of the purified protein. As shown in Figure S2, the Trp-analog-containing peptides gave strong signals clearly distinct from native Trp-containing peptides. The highest incorporation efficiency was observed for 5,6diFW (>99% at both Trp positions), followed by 5MeW, 5FW, and 5,6,7triFW with at least 95% incorporation efficiency at both Trp positions (Table 1). The incorporation efficiencies of 4,5,6,7tetraFW were >96% and 76% for positions 96 and 67, respectively (Table 1). Taken together, LmrR labeled with one of the four fluorinated Trp analogs or with 5MeW can be well expressed in our *L. lactis* expression system, yielding milligram amounts of recombinant protein.

**Table 1:** Trp Analog Incorporation Efficiency at Positions 67 and 96 in LmrR

	Incorporation efficiency	
Trp Analog	Positon 67	Position 96
5MeW	>99%	>96%
5FW	>95%	>99%
5,6diFW	>99%	>99%
5,6,7triFW	>98%	>97%
4,5,6,7tetraFW	>76%	>96%

### Binding Affinity of Dau and RBF to LmrR

**Figure 1:**
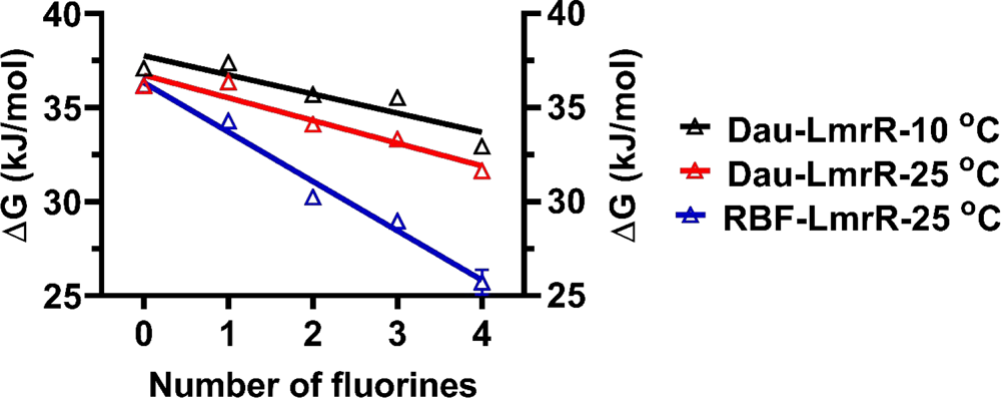
Relationship between the number of fluorine atoms in the indole group of W96 in LmrR and the binding energy (Δ*G*) upon binding of (blue) RBF at 25 °C, (red) Dau at 25 °C, and (black) Dau at 10 °C. The bars represent standard deviations (*n* = 3).

LmrR binds different drugs including Dau and RBF.^19,20^ To investigate the importance of π–π interactions for drug binding, the electron density in the π electron cloud of W96 was modulated by progressive fluorination of the W96 indole side chain. The binding affinities of LmrR for Dau and RBF decreased upon increasing degrees of fluorination of W96 (Table 2, Figure S3). For instance, at 25 °C, the affinity of LmrR for RBF decreased 70-fold, with the *k*_d_ going from 460 nM (W-LmrR) to 33 400 nM (4,5,6,7tetraFW-LmrR). Dau behaved less extreme, with the *k*_d_ decreasing 6-fold (Table 2). These results show that the drug binding affinity is very sensitive to the electron density in the π electron cloud, demonstrating a significant contribution of the electrostatics of the π–π interaction to the total LmrR-drug binding energy. As shown in Figure 1, the decrease of the free energy of binding, Δ*G*, derived from the *K*_d_ values at 25 °C, shows for both drugs a linear dependence on the number of fluorine substituents in the indole moiety.

**Table 2:** Dissociation Constants of LmrR Variants with Dau and RBF

	Binding affinity, *K*_d_ (nM) (Mean ± SD)^a^		
LmrR protein	Dau at 10 °C	Dau at 25 °C	RBF at 25 °C
5MeW	104 ± 5	370 ± 30	380 ± 8
W	315 ± 40	450 ± 50	460 ± 50
5FW	280 ± 40	430 ± 80	970 ± 50
5,6diFW	560 ± 50	1050 ± 60	4990 ± 60
5,6,7triFW	590 ± 60	1440 ± 100	8370 ± 200
4,5,6,7tetraFW	1680 ± 70	2880 ± 500	33400 ± 16000

a “SD”, standard deviation (*n* = 3).

While fluorine substitutions of Trp decrease the electrostatic potential of the indole π electron cloud, a methyl substitution increases it, as methyl groups are electron-donating.^4^ As shown in Table 2 the affinity of LmrR for the Dau and RBF drugs increases, compared to wild type, when W96 is methylated at the 5-position. When the Dau and RBF binding energies of this and the other five LmrR proteins are plotted against the *in silico* calculated cation-π interaction energies^4^ of indole and the indole analogs incorporated at position 96, also a linear free energy relationship is obtained (Figure S4). This result further supports the electrostatic contribution of π–π interactions to drug binding by LmrR.

### Dau-LmrR Binding Experiments at 10 °C

The thermodynamics of the LmrR–Dau interaction was recently reported to be mostly entropy driven.^21^ To investigate this point in more detail the temperature dependence of the LmrR–Dau interaction was studied by surface plasmon resonance (SPR) experiments at 10 and 25 °C. As shown in Table 2, a lower temperature increased the binding affinity 1.4–3.5-fold for all LmrR variants. The free energy of binding, Δ*G*, derived from the dissociation constants, is shown in Table 3. Comparing Δ*G*_10 °C_ – Δ*G*_25 °C_ for Dau shows that the ΔΔ*G* values are not significantly different from 0 kJ/mol, considering the standard deviations of typically <2% for the Δ*G* data presented in Table 3. The average ΔΔ*G* from 6 LmrR variants is around 0.1 kJ/mol. These values imply that the temperature effects on the Δ*G*s are negligible, which would be in line with a low contribution of entropic factors to the Dau–LmrR interaction.

**Table 3:** Experimental Free Energy Δ*G* of Ligand Binding to LmrR Variants and Free Energy Difference ΔΔ*G* of Dau Binding to LmrR Variants between at 10 and 25 °C

	Dau (kJ/mol)		ΔΔ*G* Dau (kJ/mol)	RBF (kJ/mol)
LmrR protein	Δ*G*_10 °C_	Δ*G*_25 °C_	Δ*G*_10 °C_ – Δ*G*_25 °C_	Δ*G*_25 °C_
5MeW	–37.8	–36.7	–1.1	–36.6
W	–35.2	–36.2	0.9	–36.2
5FW	–35.5	–36.4	0.9	–34.3
5,6diFW	–33.9	–34.1	0.2	–30.2
5,6,7triFW	–33.7	–33.3	–0.4	–29.0
4,5,6,7tetraFW	–31.3	–31.6	0.3	–25.7

The SPR assays can also inform on the kinetics of the LmrR-drug interaction, but for the assays at 25 °C, the *k*_on_ and *k*_off_ rates could not be analyzed properly, because the rates were too fast at those conditions. At 10 °C, the *k*_off_ rates could be determined, except for 5,6diFW-LmrR, and the results are presented in Table 4. The *k*_off_ rates increase when the electron density of the π-electron cloud in W96 decreases, in support of a sizable contribution of the π–π stacking interaction to the drug binding affinity.

**Table 4:** *k*_off_ Rates of Dau Binding to LmrR Variants Obtained from SPR Response Traces at 10 °C

LmrR protein	*k*_off_ (s^–1^) (Mean ± SD)^a^
5MeW	0.2 ± 0.04
W	0.4 ± 0.07
5FW	0.6 ± 0.001
5,6diFW	—b
5,6,7triFW	0.8 ± 0.1
4,5,6,7tetraFW	1.5 ± 0.09

a “SD”, standard deviation (*n* = 3).

b Data could not be determined.

The previously reported LmrR experiments that led to the conclusion that Dau binding is mostly entropy driven^21^ were conducted in a different buffer system. We noticed that LmrR, when mixed with Dau, is prone to aggregation in this buffer system. This aspect was studied in more detail using dynamic light scattering and gel filtration experiments, and these experiments are presented in the Supporting Information.

### Crystal Structures

**Figure 2:**
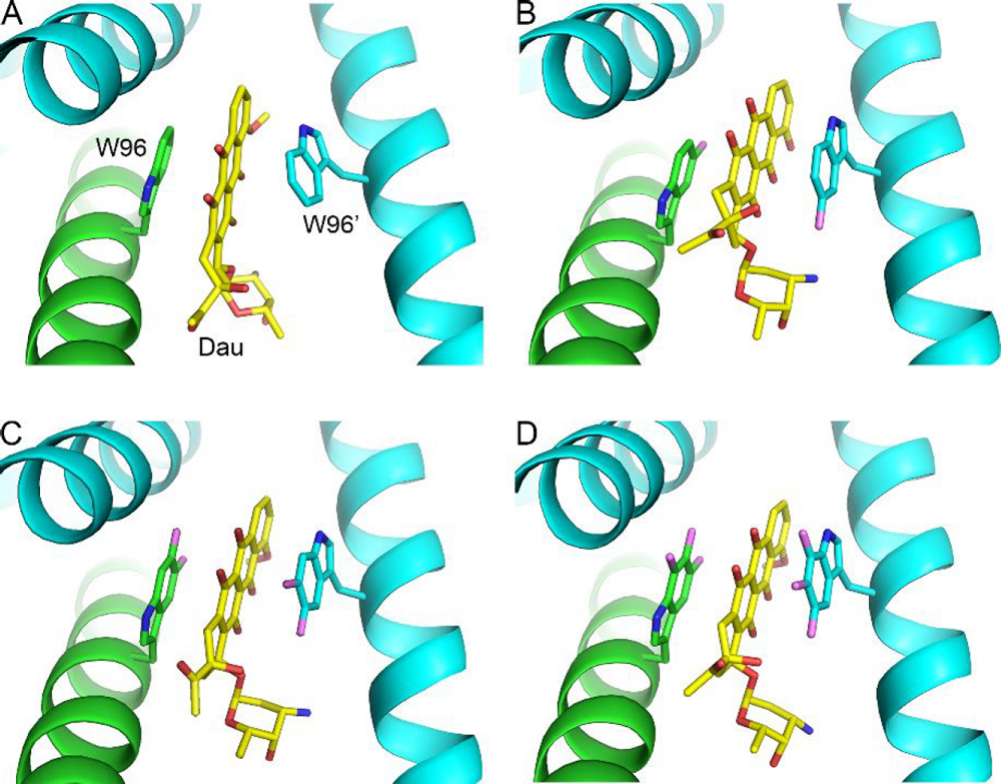
Binding modes of daunomycin in the crystal structures of wild-type LmrR and the three LmrR W96 fluoro-substituted variants. (A) Wild-type LmrR-Dau complex (PDB entry 3F8F(19)), (B) 5FW-LmrR-Dau (PDB entry 7QZ6, this work), (C) 5,6diFW-LmrR-Dau (PDB entry 7QZ8, this work), (D) 5,6,7triFW-LmrR-Dau (PDB entry 7QZ7, this work). The two chains of the LmrR dimer are colored in cyan and green. Daunomycin is colored in yellow (carbons), red (oxygens), and blue (nitrogens).

To assess whether the Trp analogs influence the overall structure of LmrR and its drug-binding pocket, crystal structures were determined of LmrR with bound Dau for LmrR variants 5FW, 5,6diFW, and 5,6,7triFW (Figure 2; Table S1). The crystal structures belong to the same crystal form (space group C2 with very similar unit cell dimensions) and were refined to resolutions varying between 2.55 and 2.15 Å. Although the crystal structures show generally good geometry, they all suffer from high crystallographic B-factors (average B-factors for protein atoms varying between ∼57 and ∼89 Å^2^, Table S1), indicative of static disorder in the crystals. Nevertheless, the electron density maps were of sufficient quality to identify the Trp analogs at positions 67 and 96 in both polypeptide chains of the dimeric LmrR variants. No major differences were observed in the backbone and side chain conformations around residues W67 and W96, compared to wild-type LmrR (Figure S5).^19^ Moreover, as shown in Figures 2 and S6, the binding mode of Dau to LmrR is not substantially affected by the presence of the fluorinated Trp analogs. Dau is bound in the same way as in the wild-type protein, with the aromatic part of Dau sandwiched between two Trp analog residues, stabilized by π–π stacking interactions. Thus, we conclude that the different Dau binding affinities observed for the LmrR variants are not related to conformational changes of the protein or significantly altered ligand binding modes.

### RibU

Next, the potential of the developed expression system was explored for progressive fluorination of W68 in the S component of the riboflavin (RBF) transporter, RibU, from *L. lactis*. This integral membrane protein of 22.8 kDa binds its cognate ligand RBF with very high affinity (*K*_d_ ≈ 1 nM).^23^ RibU has three Trp residues at positions 68, 79, and 97, and after mutating each Trp to a Tyr, only mutating W68 was found to affect the RBF binding affinity as it dropped ∼100-fold for the W68Y mutant.^23^ Additional evidence that W68 is involved in RBF binding came from fluorescence experiments as the fluorescence of W68 became completely, and RBF fluorescence almost completely, quenched upon RBF binding. This quenching allows measuring the binding affinity via fluorescence titration experiments.^23^ In the present work, the fluorination approach was applied for W68. RibU mutant W97Y could be efficiently overexpressed (Figure S7), but the yield of the purified protein was less than found for LmrR, a typical observation when comparing yields of water-soluble and membrane-bound proteins. Like for LmrR, progressive fluorination of Trp resulted in lower expression levels but enough purified RiBU was obtained for evaluating its interaction with RBF. Only for RibU labeled with 4,5,6,7-tetraFW the yield was too low (Figure S7) for a reliable determination of the RBF binding affinity.

**Figure 3:**
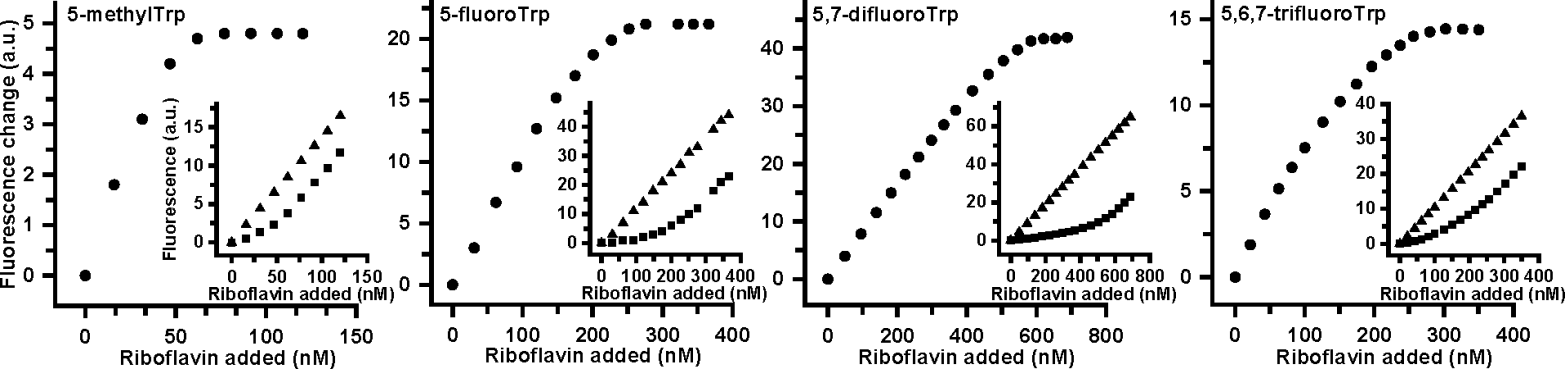
Titration of Trp analog labeled RibU W97Y proteins with RBF. The RBF fluorescence was measured at the indicated RBF concentrations in the absence (triangles) or presence (dots) of the W97Y RibU variant (see insets). The difference between these two signals were calculated to build the titration curve. This curve was used to compute the RBF *K*_d_ (see Experimental Section in the Supporting Information). Panels from left to right present the results for RibU W97Y, labeled with 5MeW, 5FW, 5,7diFW, and 5,6,7triFW, respectively.

Fluorescence titration experiments of the different RibU constructs with RBF at 20 °C allowed estimation of the impact of W68 fluorination or 5-methylation on the binding affinity (Figure 3 and Table 5). Progressive fluorination of W68 resulted in a gradual 15-fold lowering of the affinity when up to three fluoro-atoms are introduced, while the affinity is somewhat enhanced for RibU labeled with 5-MeW.

**Table 5:** Dissociation Constants of RibU Variants with RBF at 20°C

RibU variants	Binding affinity, *K*_d_ (nM) (Mean ± SD)^a^
5MeW	1.2 ± 0.1 (*n* = 2)
W	1.8 ± 0.8 (*n* = 3)
5FW	5.4 ± 1.0 (*n* = 4)
5,7diFW	8.2 ± 0.9 (*n* = 3)
5,6,7triFW	24.9 ± 2.7 (*n* = 2)

a “SD”, standard deviation.

**Figure 4:**
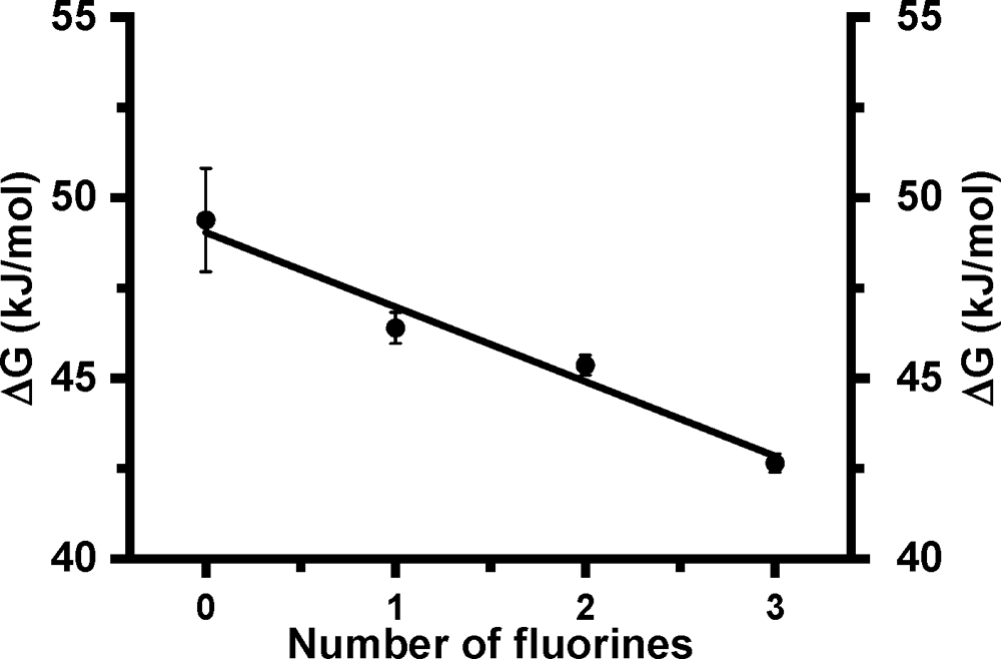
Relationship between the number of fluorine atoms in the indole group of W68 in RibU and the released binding energy (Δ*G*) upon binding of RBF at 20 °C. The bars represent standard deviations (*n* = 2–4).

Converting the *K*_d_’s into the free energy of binding, Δ*G*, and plotting Δ*G* against the number of fluorine atoms in W68 yielded a linear free energy relationship (Figure 4). A similar relation is obtained when Δ*G*’s of all five RibU variants are plotted against the *in silico* calculated cation−π interaction energy^4^ (Figure S8). The linear free energy relationships support the view that an electrostatic component of W68 makes a significant contribution to RBF binding at RibU.

## Discussion

The work presented here has yielded a number of significant advances. First, we developed an efficient expression system for producing milligram quantities of proteins labeled with an isosteric Trp analog bearing one to four fluorine atoms or a methyl group on the indole moiety. Second, by incorporating various Trp analogs we could quantitate the electrostatic contribution of π interactions to LmrR–drug and RibU–riboflavin complex formation.

### Expression System for Producing Mg Quantities of Proteins Labeled with a Fluorinated or Methylated Trp Analog

Biosynthetic incorporation of Trp analogs using a Trp auxotroph expression system is a well-established methodology used in many different laboratories.^24−28^ While monofluorinated Trp analogs can readily be incorporated in proteins using an auxotrophic strain,^29^ the restricted substrate specificity of tryptophanyl tRNA synthethase has so far prevented the incorporation of Trp analogs with a higher degree of fluorination. The same is true for multifluorinated analogs of Tyr and Phe, when using a Tyr or Phe auxotrophic expression system, respectively. Multifluorinated Trp analogs are excellent probes for investigating the role of Trp in noncovalent interactions, like cation−π or π–π interactions, as demonstrated by the pioneering work of the Dougherty group using their oocyte expression platform.^3,13,30−32^ In this system, amber suppressor tRNA is chemically acylated with a fluorinated analog and the adduct is injected in an oocyte cell together with the mRNA of the target protein, mutated with an in frame amber codon. The attractive feature of this expression system is the site-specific labeling of the target protein with an unnatural amino acid, but a major limitation is the extremely low yield of labeled protein, typically in the order of picomole quantities.^9^ Nevertheless, the electrophysiological characterization of fluorine-labeled proteins has been exceptionally successful for several important neurobiological ligand-gated-ion channels like the serotonergic and nicotinic acetylcholine receptors.^12^

Genetic encoding using orthogonal tRNA/tRNA synthetase pairs is another approach to site-specifically incorporate unnatural amino acids into proteins. This was used for the *in vivo* incorporation of fluorinated Phe residues or Phe residues para-labeled with a CH_3_, CF_3_, Cl, CN, or NO_2_ group.^33−36^ However, the incorporation of fluoroPhe analogs showed limited fidelity, resulting from contamination by the structurally very similar unlabeled amino acid.^36^ The para-substituted Phe analogs could only be incorporated if these quite large substituents do sterically fit in the protein structure. Thus, there is interest in high yield expression systems that produce, with high specificity, fluoroTrp labeled proteins.

Here, we have reported an *L. lactis* Trp auxotroph expression system, which thanks to the coexpression of the *L. lactis* tryptophanyl-tRNA synthetase is able to produce milligram amounts of proteins labeled with a fluorinated or methylated Trp analog. The system can be easily employed in a standard equipped biochemical laboratory and is not very complicated, as it uses a synthetic expression medium consisting of maximally 30 components.^37^*L. lactis* is a fast-growing organism that can achieve high cell densities under both aerobic and anaerobic conditions, for which well-regulated promotor expression systems are available, and in which no inclusion bodies are formed. It has been used extensively for the functional overexpression of soluble and membrane-bound proteins originating from different kingdoms.^38^ We obtained milligram quantities of pure, labeled protein, which allowed, for the first time, the crystallization and structure determination of a set of proteins labeled with mono-, di-, or trifluoroTrp. It provided detailed insights into the effects of fluorination on the binding interactions of the indole side chain (Figures 2, S5, and S6). By our method, all Trp residues that are present in a protein are replaced by a Trp analog, thus making the system most suitable for proteins containing a limited number of Trp residues. Trp is the least abundant amino acid in proteins, but compared to the other aromatic residues, its indole π electrons are most often involved in noncovalent interactions.^8,39,40^ This makes our expression system suitable to investigate these interactions in many different protein systems. For example, there is increasing interest in the role of Trp in carbohydrate–protein (CH−π) interactions, which may play a pivotal role in the specificity and binding affinity of the interaction.^8,41−43^ To date, quantitative data of this interaction in proteins are not available.^44^

### Role of W96 in LmrR–Drug Interaction

3D structures of LmrR with bound Hoechst33342, Dau, and RBF have provided detailed insights into how the structurally different ligands are recognized (Figure S1).^19,20^ The drugs bind with their aromatic group between the side chains of W96 and W96′ (residue 96 from the other monomer), which are located face-to-face at ∼7 Å from each other in the center of the drug-binding site.^19^ LmrR binds the drugs Dau and RBF with an affinity of 450 and 460 nM, respectively (see Table 2), in agreement with the results of Takeuchi et al., who also used SPR to measure affinity.^21^ Binding of Dau and RBF to LmrR occurs via π–π stacking interactions, which allows the binding of a wide diversity of aromatic compounds,^19^ but the contribution of the π–π stacking interactions to the affinity could not be determined. By labeling LmrR with fluorinated and methylated Trp analogues, we find that in LmrR the electrostatic component of π–π interactions has an important contribution to the binding affinity, as illustrated by Figures 1 and S4. It clearly shows that the binding energy decreases with an increasing number of electron-withdrawing fluorine atoms in the Trp side chain. Thus, electrostatic interactions are important for drug binding by LmrR.

### Magnitude of the π–π Stacking Energy When LmrR Binds Dau or RBF

As can be seen from Figure 1, the free energy of binding of the drugs, Δ*G*, is linearly dependent on the number of fluorine substituents. Since four fluorine substituents in a Trp completely remove the negative electrostatic potential on the face of the aromatic ring,^9,13^ the electrostatic contribution of 4,5,6,7tetraFW to the π–π interactions will be about zero. Thus, 4,5,6,7tetraFW can serve as a baseline to obtain the electrostatic contributions of π–π interactions to the total binding energy. Because of the linear relationship of binding energy and number of fluorine substituents, and the absence of significant conformational differences between the analog-labeled LmrR proteins, a straightforward extrapolation to the situation with zero fluorine substituents (wild type Trp at position 96) can be made. Thus, we obtain, as contributions of the π–π interactions to the total binding energy at 25 °C, values of −4.8 and −10.5 kJ/mol for Dau and RBF, respectively, which correspond to 13% and 29% of the total binding energy, respectively. With this contribution, a 6- and 70-fold enhancement of the binding affinity was obtained for Dau and RBF, respectively, making π–π stacking a sizable contribution to the binding of aromatic compounds by LmrR. However, the importance of the π–π interactions is different for the two drugs, which is likely related to their electrostatic potential. For Dau, the electrostatic potential of the aromatic ring system is neither high nor low^45^ limiting the stacking energy potential. Approximately half of the aromatic surface of the isoalloxazine ring of RBF shows a positive electrostatic potential, complementary to the negative electrostatic potential of Trp, while in the other half the electrostatic potential is close to neutral.^46^ Still, for both compounds, the contribution of the π–π stacking energy to the overall binding energy was found to be significant. Our work predicts that drugs containing an aromatic ring system with a positive electrostatic potential will bind most strongly to LmrR, as long as the drug can sterically fit in the binding site without experiencing repulsive interactions.

### Entropy Contribution to the LmrR–Dau Interaction

Data of binding experiments of LmrR and a drug at different temperatures can potentially inform about the contribution of entropy to Δ*G*. Data presented in Table 2 indicate that the temperature does not significantly affect the binding affinity of Dau. All *K*_d_ values decrease when the temperature is decreased from 25 to 10 °C (Table 2), but the associated Δ*G* values remain fairly constant (Table 3). The slopes of the lines in the fluorination plots (Figure 1) are also similar, indicating that the strength of the π–π interaction is not strongly temperature dependent. The insensitivity of Δ*G* to temperature suggests, but does not prove, that the entropy contribution to Δ*G* is small. This conclusion is inconsistent with the results of Takeuchi et al., who concluded that binding of drugs to LmrR is entropy driven, as an increase in conformational flexibility was observed upon drug binding. This increase in flexibility was estimated as 2–6 kJ/mol contributing to the binding energy, Δ*G*. Although significant, this contribution is at the lower range of what we obtained for the stacking energy contribution to Δ*G*. Complementary ITC experiments suggested that the enthalpic contribution to Δ*G* is small or unfavorable. For example, ITC experiments with LmrR and Dau showed the absence of an enthalpic energy contribution to Δ*G*.^21^ However, the conditions at which the ITC experiments were done led to aggregation in our experiments (see Supporting Information and Figures S9 and S10), which precludes inferring firm conclusions on the enthalpic contributions. Taken together, SPR data of the LmrR–Dau interaction collected at 10 and 25 °C do suggest that the Δ*G* and the strength of the π–π stacking interaction are minimally dependent on temperature. This makes it unlikely that this interaction is essentially entropy driven.

### Role of W68 in RibU–RBF Interaction

High resolution structural information about membrane transport proteins has increased significantly in the past decade, and as a result solute transport mechanisms have been elucidated at the molecular level for many different transport proteins. The dynamics of noncovalent binding interactions within the protein structure and between protein and substrate form the basis of the transport cycle, and this dynamical process is energized by a chemical process like ATP hydrolysis or chemical gradient energy. A role for noncovalent interactions involving π electrons during the transport process has been proposed but have not been quantified or rigorously evaluated to date.^47−49^ While many transporters bind their substrate with low affinity, others show dissociation constants in the pM–nM concentration range. The class of transporters with the highest substrate binding affinity belong to the energy-coupling factor (ECF)-type ATP-binding cassette (ABC) transporters.^50^ They are responsible for the uptake of vitamins and micronutrients, and the low *in vivo* concentrations of these substrates explain the need for a transporter with a high binding affinity. Substrate is bound by an integral membrane subunit, called the S component. The 3D structures of multiple members of these transporters have been reported in the absence and presence of substrate. In the S component substrate complexes, typically all possible H-bonding sites between ligand and transporter are occupied, as well as all ionic interactions, rationalizing the extremely high binding affinity.^50^ However, aromatic residues are also present in close contact with the substrate.^50^ Their impact on the binding affinity has been investigated most extensively for the S component of the riboflavin (RBF) transporter, RibU, from *L. lactis*, and W68 was found critical for RBF binding.^23^ In the present work, the fluorination approach was applied for W68 and our results show that the affinity of RibU for RBF drops sharply upon progressive fluorination of W68 (Table 5). A good correlation was obtained when the free energy of RBF binding was plotted against the number of fluorine atoms in W68 (Figure 4) or against the *in silico* calculated cation−π interaction of the Trp analog (Figure S8). Extrapolation to RibU labeled with tetrafluoroTrp yields an ∼8 kJ/mol lower Δ*G* for RBF binding, a value corresponding to 15–20% of total RBF binding energy of RibU containing natural Trp.

The 3D structure of RibU from *L. lactis* is not known, but in the structures of the S component substrate complexes the residue equivalent to Trp68 of RibU is at the end of helix 3 and points toward the substrate binding pocket.^50−53^ More than a dozen residues in the S component of two RibU homologues, of which structures were solved, are directly involved in substrate binding.^51,52^ The 15–20% of total RBF binding energy by RibU that we attribute to W68 highlights the importance of this single residue for generating nanomolar affinity for RBF.

## Conclusion

In this work we present a new protein expression system for progressive tryptophan fluorination studies. It features an excellent Trp analog incorporation efficiency and high protein yield, permitting in depth studies of the role of π interactions for which milligram amounts of sample are required. This system complements the oocyte expression system for which an electrophysiological readout is needed to analyze the labeled proteins. The *L. lactis* expression system can be easily employed in a standard equipped biochemical laboratory. It has the potential to obtain detailed information on the role of π interactions in many different fields of protein chemistry. In a first example, we quantified the π–π bonding interaction between the LmrR protein and two of its ligands, showing the sizable contribution of the π–π interactions to the ligand affinity. The availability of experimental quantitative data on π interactions will not only deepen our fundamental knowledge about these interactions but also help improve theoretical approaches to calculate the energy of these interactions. Progress in protein science relies increasingly on protein structure and protein activity predicting algorithms. Expanding our knowledge about these ubiquitous noncovalent interactions in proteins may permit addition of a sophisticated algorithm to the tool boxes currently in use in programs of *de novo* protein design, rational drug design, and rational biobased-material design.

## Supporting Information Available

Figures S1–S10, Table S1, Solubility of LmrR and LmrR-Dau/RBF complexes in phosphate buffer, Experimental Section, and References (PDF)
