# Supplementary material for: The Role of Tryptophan in π Interactions in Proteins: An Experimental Approach
Source: J Am Chem Soc. 2022 Jul 22;144(30):13815–22. doi: 10.1021/jacs.2c04986 (PMC9354243; doi:10.1021/jacs.2c04986)
Supplement: Supplementary file 1 — ja2c04986_si_001.pdf [file ja2c04986_si_001.pdf]

The role of tryptophan in π interactions in proteins: An experimental approach

Jinfeng Shao[1], Bastiaan P. Kuiper[1], Andy-Mark W. H. Thunnissen[1], Robbert H. Cool[2], Liang Zhou[1], Chenxi Huang[1], Bauke W. Dijkstra[1], and Jaap Broos[1*]

[1]Groningen Biomolecular Science and Biotechnology Institute (GBB), University of Groningen, Nijenborgh 7, 9747 AG Groningen, The Netherlands

[2] Department of Chemical and Pharmaceutical Biology, University of Groningen, Antonius Deusinglaan 1, 9713 AV Groningen, The Netherlands

[*]Email: J.broos@rug.nl

Supporting Information

Table of Content **Page**

Section	Page
**Figures S1-S10**	**2-9**
**Table S1**	**10**
**Solubility of LmrR and LmrR-Dau/RBF complexes in phosphate buffer**	**11**
**Experimental Section**	**11-17**
**References**	**17-18**

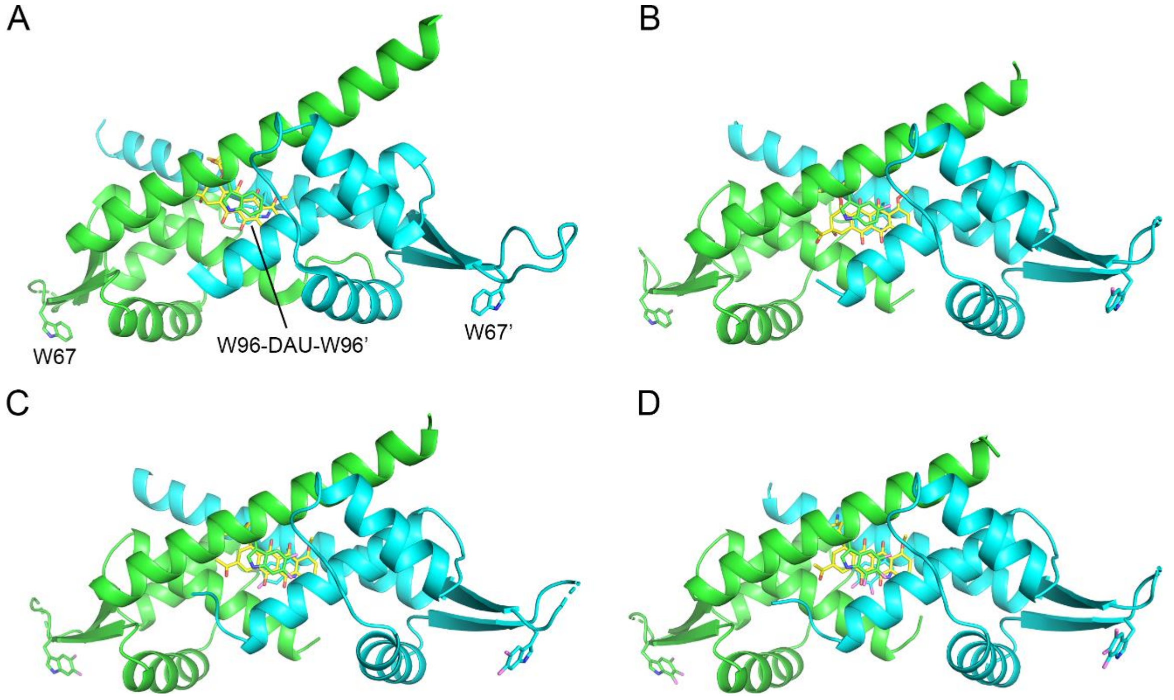

Figure S1. Overall structures of wild-type LmrR and the fluorinated LmrR variants with bound daunomycin, also showing the side chains of the fluoro-substituted tryptophans. (A) Wild-type LmrR with bound Dau (PDB entry 3F8F).[1] (B) LmrR-5FW with bound Dau (PDB entry 7QZ6, this work), (C) 5,6,diFW-LmrR with bound Dau (PDB entry 7QZ8, this work), (D) 5,6,7,triFW-LmrR with bound Dau (PDB entry 7QZ7, this work). The two chains of the LmrR dimer are colored in cyan and green. Dau is colored in yellow (carbons), red (oxygens) and blue (nitrogens).

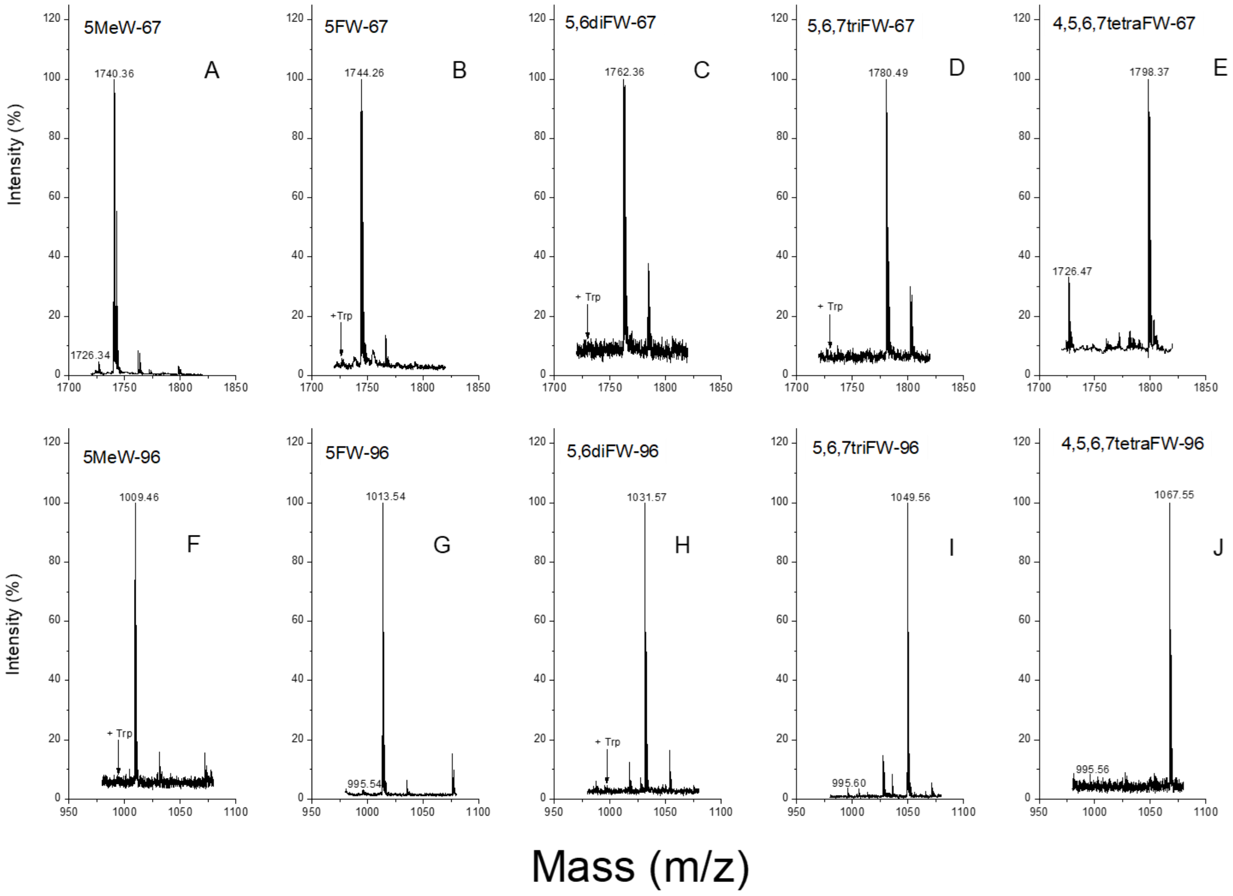

Figure S2. MALDI-TOF spectra of tryptic peptides of LmrR containing Trp or a Trp analog at positions 67 and 96. (A-E) Peptide DGIISSYW^{67}GDESQGGR (theoretical m/z 1726.8 Da) containing 5MeW (A), 5FW (B), 5,6diFW (C), 5,6,7triFW (D) and 4,5,6,7tetraFW (E), are presented, respectively. (F-J) Peptide LAFESW^{96}SR (theoretical m/z 995.1 Da) containing 5MeW (F), 5FW (G), 5,6diFW (H), 5,6,7triFW (I) and 4,5,6,7tetraFW (J), are presented, respectively. The position of the native Trp-containing peptide, when detectable, is also indicated.

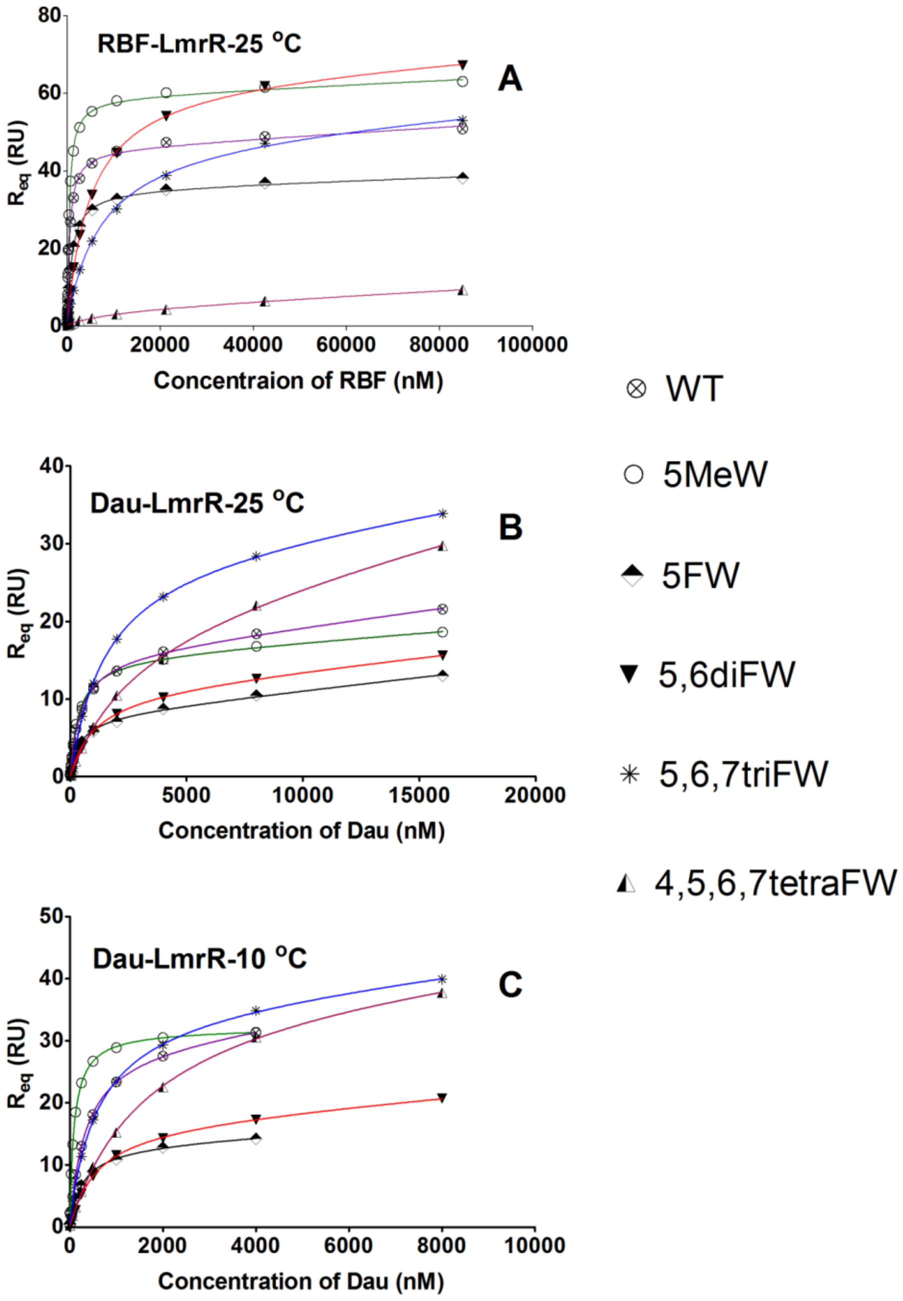

Figure S3. SPR titration curves of (A) RBF at 25 °C, (B) Dau at 25 °C, and (C) Dau at 10 °C, binding to various LmrR variants. The Y axis shows the response of the ligand at the equilibrium state (in response units (RU)). The X axis shows the concentration of ligand used for each titration. Different symbols were used to show the different Trp-analog-labeled-LmrR proteins.

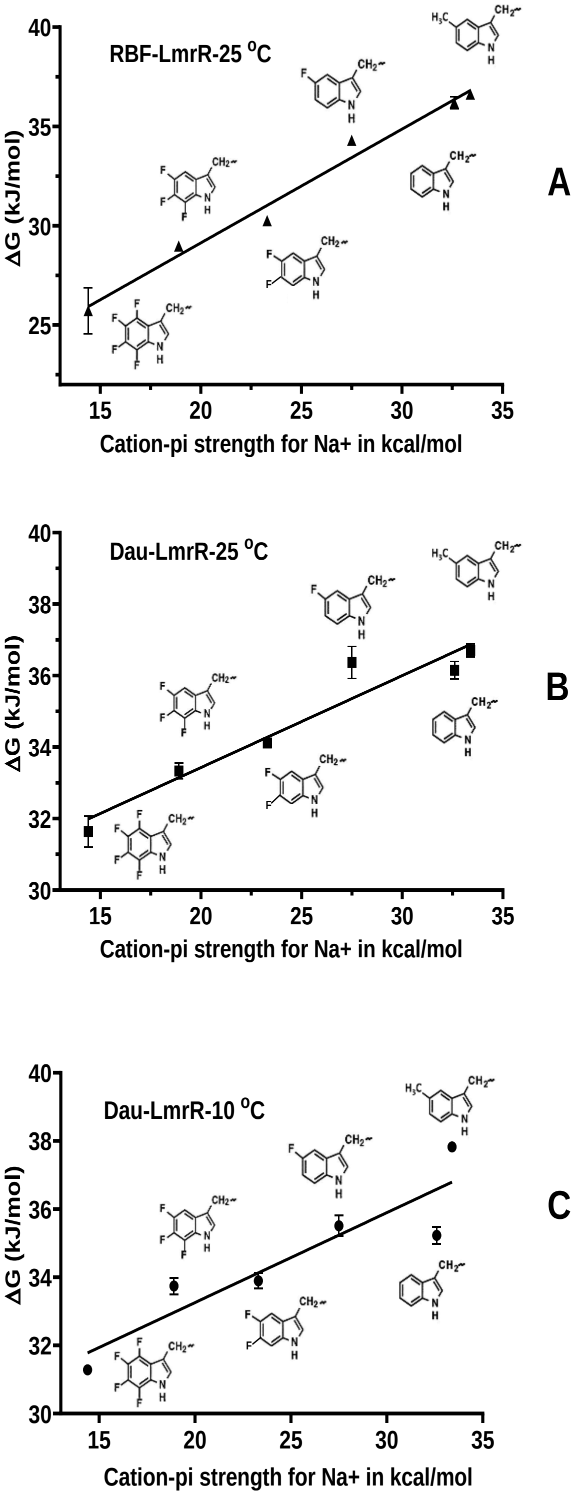

Figure S4. Relationship between the *in silico* calculated cation-π binding energy[2] and the binding energy (ΔG) of LmrR mutants, labeled with different indole moieties, toward RBF or Dau. (A) Data for RBF binding to LmrR mutants at 25 °C. (B) Data for Dau binding to LmrR mutants at 25 °C and (C) Data for Dau binding to LmrR mutants at 10 °C. The bars represent standard deviations (n=3).

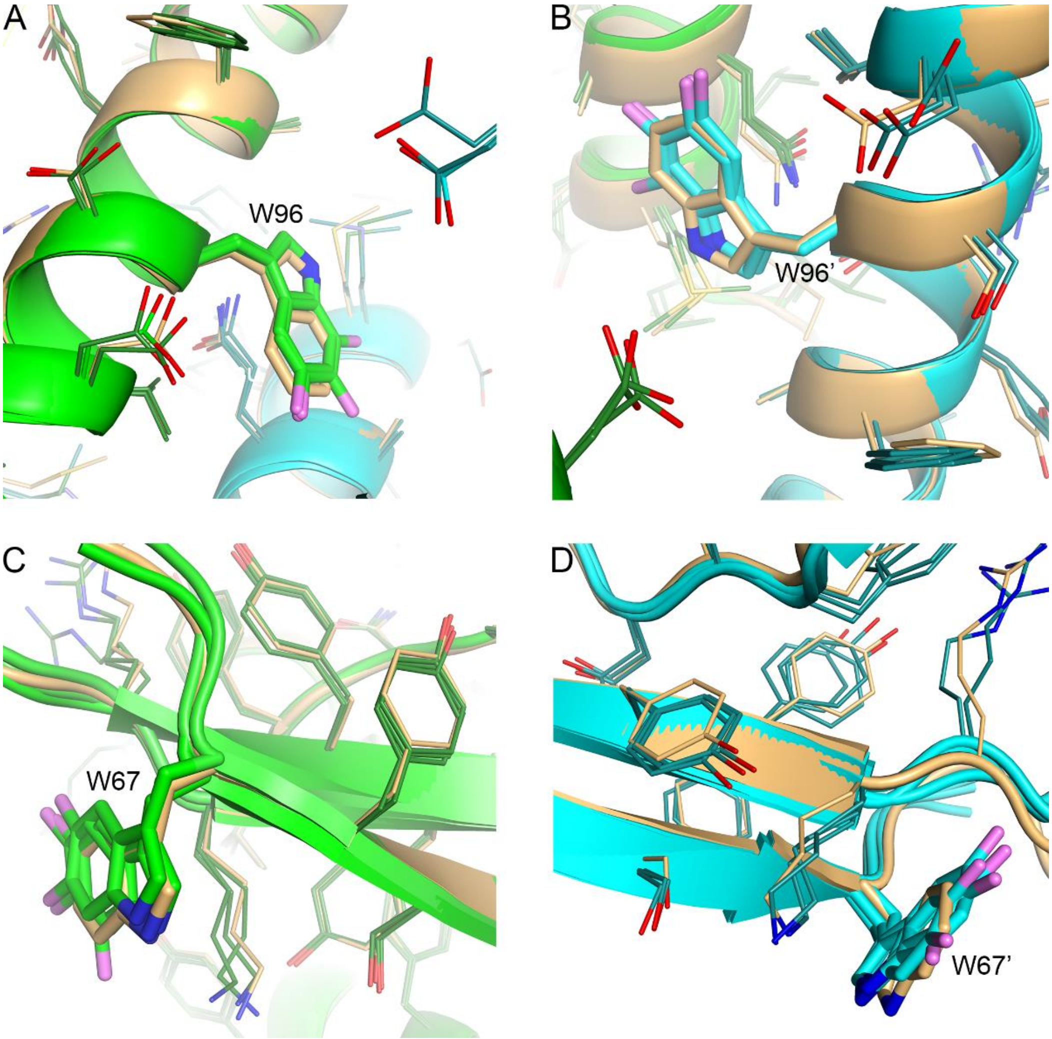

Figure S5. Overlays of the crystal structures of wild-type LmrR and the fluorinated LmrR variants. The panels display zoomed-in views of the Trp and Trp analogs at positions 96/96' and 67/67' in the LmrR dimers (the apostrophe denotes that the residue belongs to the opposite chain), including the side chains of surrounding residues**.** (A,B) Residues 96 and 96' in the LmrR dimers. (C,D) Residue 67 and 67' in the LmrR dimers. The two chains of the LmrR variants are colored in green and cyan, the chains of the wild-type LmrR dimer are shown with a single color (wheat). Coordinates of wild-type LmrR are from PDB entry 3F8F.[1] Coordinates of the LmrR variants are from PDB entries 7QZ6, 7QZ7, and 7QZ8 (this work). For clarity, daunomycin is not shown in panels A and B.

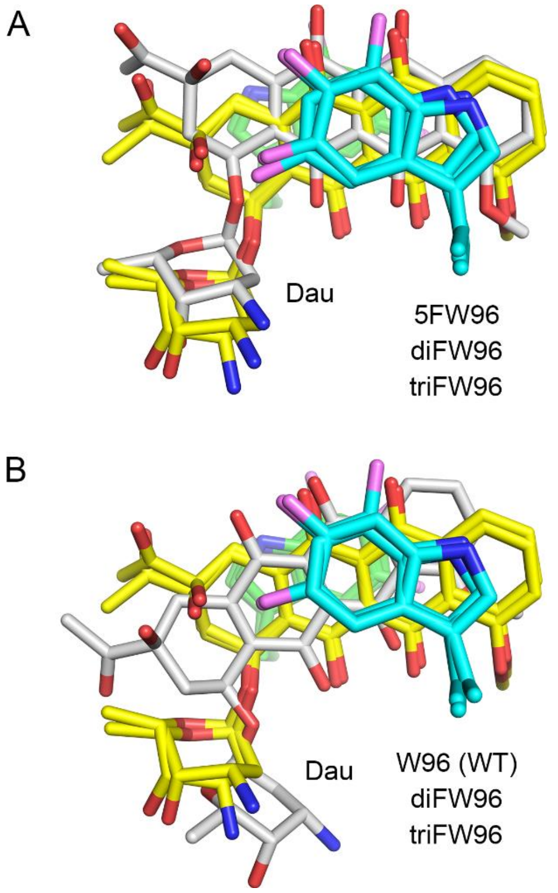

Figure S6. Comparison of the Dau binding modes in the different LmrR fluorinated variants. (A) Overlay of 5FW-LmrR-Dau, 5,6-diFW96-LmrR-Dau and 5,6,7triFW-LmrR-Dau. (B) Overlay of wild-type LmrR-Dau, 5,6-diFW96-LmrR-Dau and 5,6,7triFW-LmrR-Dau. The Dau molecules interacting with fluorinated tryptophan residues 96 and 96’ in 5,6diFW-LmrR and in 5,6,7triFW-LmrR are colored with yellow carbons, while the Dau molecules bound in 5FW-LmrR and wild-type LmrR are colored with gray carbon atoms.

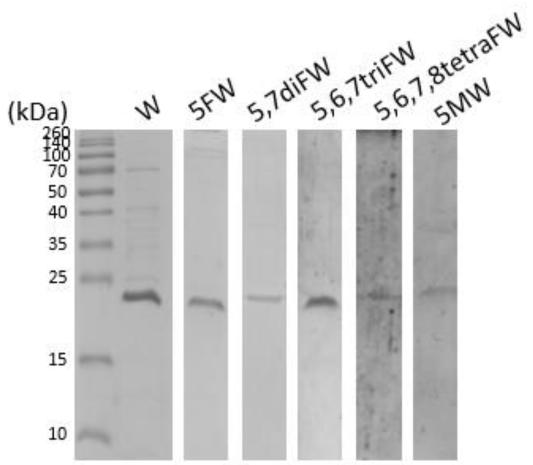

Figure S7. SDS_PAGE gels stained with Coomassie brilliant Blue of RibU fractions eluted from the gel filtration column. Lanes from left to right: molecular weight marker, purified RibU expressed in the presence of Trp (W), 5-fluorotryptophan (5FW), 5,7 difluorotryptophan (5,7diFW), 5,6,7 trifluorotryptophan (5,6,7triFW), 5,6,7,8 tetrafluorotryptophan (5,6,7,8tetraFW), and 5-methyltryptophan (5MeW).

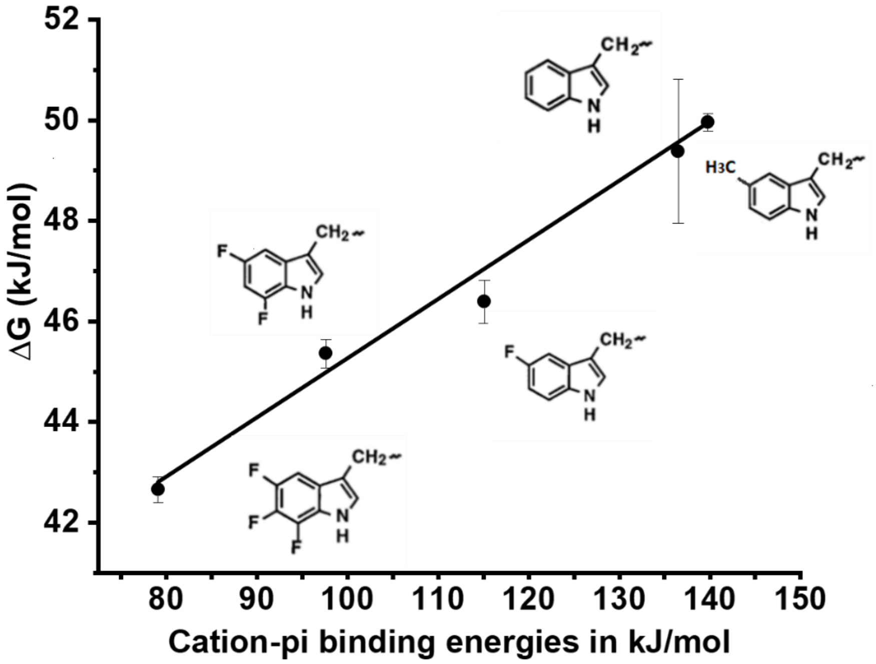

Figure S8. Relationship between the *in silico* calculated cation-π binding energy[2] and the binding energy (ΔG) of RBF at RibU, labeled with different indole moieties at 20 °C. The bars represent standard deviations (n=2-4).

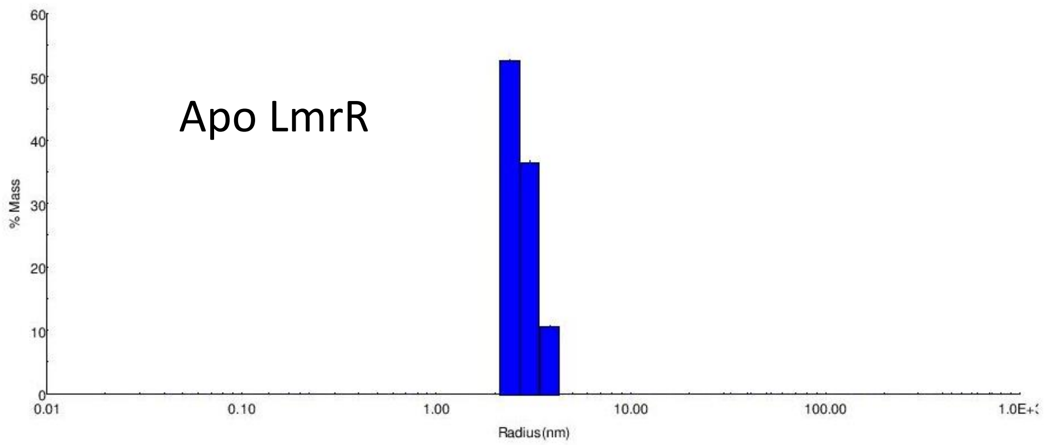

Figure S9. Dynamic light scattering profile of apo-LmrR. The hydrodynamic radius, apparent molecular weight, and polydispersity of the protein sample are 2.8 nm, 37 kDa and 17%, respectively.

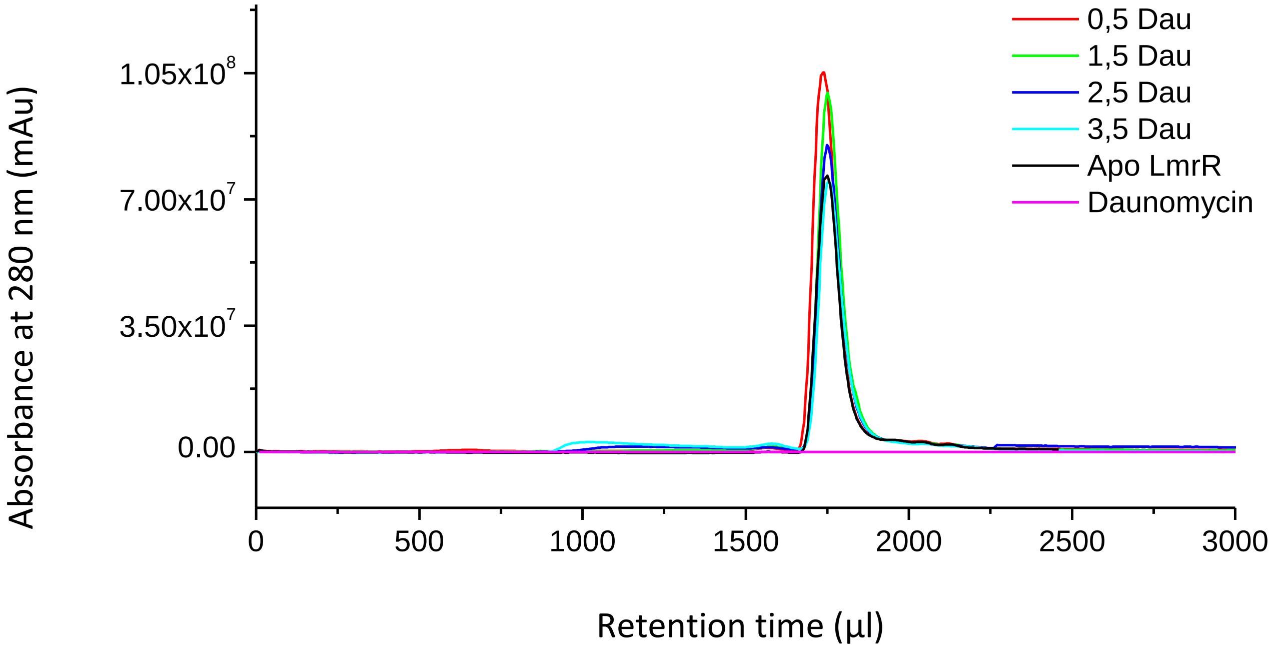

Figure S10. Gel filtration profiles of LmrR-Dau mixtures at different ratios. Some higher molecular weight species are visible at higher Dau ratios. Apo LmrR and free Dau are shown in black and pink, respectively. The molar ratios of Dau to LmrR are indicated by the numbers and different colors.

Table S1. Summary of X-ray crystallographic statistics for the fluoro-tryptophan substituted LmrR structures

	5FW	DiFW	5FW-DAU	DiFW-DAU	TriFW-DAU
Data collection					
Beamline	ID23-1	ID23-1	ID29	P13	ID23-1
Space group	C2	C2	C2	C2	C2
Unit cell dimensions, a, b, c (Å)	103.6, 35.5, 70.9	102.4, 35.0, 68.1	103.2, 35.4, 67.8	104.1, 35.7, 71.2	102.5, 35.1, 68.1
β (˚)	95.2	97.5	97.3	97.7	98.0
Resolution range (Å)[a]	44-1.80 (1.84-1.80)	43-2.31 (2.39-2.31)	44-2.15 (2.22-2.15)	70-2.15 (2.21-2.15)	34-2.30 (2.38-2.30)
Completeness (%)[a]	98.9 (94.8)	98.1 (98.2)	99.5 (99.71)	98.7 (94.2)	98.6 (98.9)
Multiplicity [a]	3.1 (3.0)	3.4 (3.5)	3.4 (3.4)	3.1 (3.1)	2.9 (3.0)
<I/sigma>[a]	8.3 (1.1)	9.0 (0.6)	14.1 (1.7)	8.7 (0.7)	9.1 (1.1)
R-merge[a]	0.032 (1.041)	0.041 (1.463)	0.038 (0.638)	0.052 (1.132)	0.048 (0.865)
R-pim[a]	0.022 (0.686)	0.026 (0.895)	0.025 (0.401)	0.036 (0.771)	0.034 (0.599)
$CC_{1/2}$[a]	0.999 (0.445)	1.000 (0.415)	0.999 (0.480)	0.978 (0.431)	0.998 (0.509)
Refinement					
Resolution range (Å)	34-1.80	38-2.31	44-2.15	52-2.30	34-2.30
R-work, R-free (%)[b]	0.217, 0.249	0.229, 0.289	0.226, 0.286	0.240, 0.324	0.224, 0.291
Protein residues: chain A	4-113	4-69, 74-112	5-68, 73-112	5-69, 73-112	4-69, 74-109
chain B	2-68, 76-113	5-68, 74-110	5-69, 74-112	3-67, 77-112	3-69, 74-108
Nr of non-H atoms: protein, waters, ligands	1803, 44, 0	1737, 0, 0	1712, 22, 66	1735, 13, 66	1717, 2, 67
Average B-factor (Å^2): protein, waters, ligands	56.6, 49.2	85.9	83.2, 60.6, 111.8	73.6, 61.9, 103.8	88.7, 65.2, 106.7
RMSD bonds (Å), angles (˚)	0.007, 0.87	0.008, 0.92	0.009, 0.93	0.007, 0.84	0.010, 0.98
Ramachandran favored (%), outl	100.0, 0.0	98.9, 0.0	98.4, 0.0	100, 0.0	100.0, 0.0
Clash score	3.99	3.55	5.43	6.39	3.89
PDB entry	7QZ5	7QZ9	7QZ6	7QZ8	7QZ7

[a] Values in parentheses refer to data in the highest resolution shell

[b] R-free is the R-factor calculated with 5% of the reflections excluded from the refinement for cross-validation. R-work is the R-factor calculated with the reflections used in the refinement.

Solubility of LmrR and LmrR-Dau/RBF complexes in phosphate buffer. In this study SPR has been used to measure the impact of W96 fluorination and methylation on drug binding affinity of LmrR. SPR was also used in the LmrR study of Takeuchi *et al.*[3] and in both studies LmrR was immobilized on a SPR chip and the same SPR buffer system was used in both studies, namely 10 mM HEPES, pH 7.4, 150 mM NaCl, 50 µM EDTA, and 0.005% surfactant P-20. The SPR results are in agreement with each other, in Takeuchi *et al.* for the drug Dau a k_d= 350 nM at 25 °C was reported, while in this study k_d= 315 nM at 10 °C and k_d= 450 nM at 25 °C (Table 2). Their conclusion that the drug binding is entropy driven is based on NMR and isothermal calorimetry (ITC) experiments conducted in 10 mM sodium phosphate buffer, pH 6.8, 100 mM NaCl using LmrR concentrations of 2.7- 5.4 mg/mL (NMR) and 0.14-1.4 mg/mL) (ITC). To assess the solubility and oligomeric state of apo and holo LmrR in this buffer, the LmrR protein was analyzed by Dynamic Light Scattering (DLS) and gel filtration as described in the Experimental section. With DLS, apo LmrR (~1 mg/ml) showed up as a monodisperse dimer (Figure S9), while no stable signal was obtained from the LmrR-RBF and LmrR/Dau (in a 1 : 10 molar ratio) complexes, indicating that ligand binding interfered with the homogeneity of LmrR. Control experiments with only the drug in the cuvette gave no scattering signal from large molecules. To further analyze the solution properties of LmrR, gel filtration experiments were carried out. A mixture of LmrR and Dau in several different molar ratios (1:0-1:3.5) eluted as a symmetric peak at about 30 KDa (elution volume around 1750 µl; Figure S10), which corresponds to the molecular weight of the LmrR dimer. Increasing the amount of Dau, yielded some higher molecular weight LmrR complexes eluting before the LmrR dimer (elution volume 900 µl; Figure S10), at ratios of 2.5 and 3.5 of Dau to LmrR, respectively. The amount of higher molecular weight LmrR is much less than the amount of LmrR dimer, but it is positively correlated with the concentration of Dau. In contrast, apo LmrR did not show any high molecular weight population (Figure S10). Taken together, the DLS and gel filtration results indicate that ligand binding influences the homogeneity of LmrR and leads to a small amount of protein aggregation at the tested buffer condition, although the data do not allow to quantitate the size and amount of the aggregates.

Experimental Section

Chemicals. Daunomycin (Dau, Calbiochem) and riboflavin (RBF, Sigma) were purchased and used without further purification. 5-fluoro-DL-Trp, 5-methyl-DL-Trp, 5,6-difluoroindole, and 4,5,6,7-tetrafluoroindole were purchased from Sigma. 6-bromo-2,3,4-trifluoroaniline was from Apollo Scientific. M17 broth was from BD Difco.

Synthesis of 5,6,7-trifluoroindole. 5,6,7-trifluoroindole was synthesizes according to the scheme shown in Chart 1.

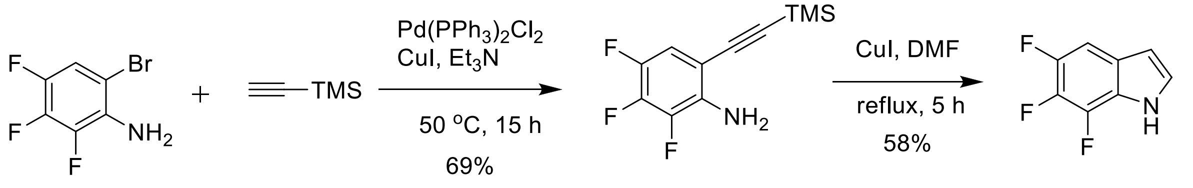

A **B** **C**

Chart 1: Synthesis of 5,6,7-trifluoro-1H-indole (**C**) from 6-bromo-2,3,4-trifluoroaniline (**A**).

To a solution of 6-bromo-2,3,4-trifluoroaniline (**A**, 1.0 g, 4.4 mmol) in triethylamine (10 mL) were added ethynyltrimethylsilane (1.56 mL, 11.0 mmol), bis-(triphenylphosphine)-palladium(II) dichloride

(46 mg, 1.5% eq) and CuI (12.5 mg, 1.5% eq). The reaction mixture was stirred for 15 hours at 50 °C under a nitrogen atmosphere. After completion, the reaction mixture was filtered using celite. The solvent was removed, and the crude product was purified by flash chromatography (5% dichloromethane in pentane) to afford 2,3,4-trifluoro-6-((trimethylsilyl)ethynyl) aniline (**B**) in 69% yield (735 mg), which was used in the following step. **B** (250 mg, 1.03 mmol) was dissolved in 7 mL DMF. After purging the solution with argon for two minutes, CuI (12 mg, 5% eq) was added. The reaction mixture was then refluxed for 5 hours. After completion of the reaction, the mixture was diluted with ethyl acetate, and washed with water. The solvent was removed, and the crude product was purified by flash chromatography (5% ethyl acetate in pentane) to afford 5,6,7-trifluoro-1H-indole (**C**) in 58% yield (100 mg) as a white crystalline solid. m.p. 40 – 45 °C; ^{1}H NMR (400 MHz, $CDCl_3$) δ 8.33 (s, br, 1H), 7.26 (s, 1H), 7.17 (d, J = 8 Hz, 1H), 6.53 (d, J = 4 Hz, 1H). ^{13}C NMR (101 MHz, $CDCl_3$) δ 147.0 (dd, J = 239.0, 12.3 Hz), 138.0 (ddd, J = 247.7, 13.5, 4.5 Hz), 136.5 (ddd, J = 241.6, 18.6, 12.3 Hz), 126.4 (d, J = 3.0 Hz), 123.5 (dd, J = 8.7, 5.0 Hz), 120.8 (d, J = 12.2 Hz), 103.6 – 103.5 (m), 101.9 (dd, J = 19.4, 3.9 Hz). ^{19}F NMR (376 MHz, $CDCl_3$) δ –145.2 (dd, J = 20.3, 10.1 Hz), –156.9 (d, J = 19.5 Hz), –169.2 (td, J = 19.9, 6.4 Hz). IR (cm−1): 3472, 3140, 1597, 1469, 1372, 1047, 947.

Synthesis and purification of Trp analogs. 5,6-difluoro-L-Trp (5,6diFW), 5,7-difluoro-L-Trp (5,7diFW), 5,6,7-trifluoro-L-Trp (5,6,7triFW) and 4,5,6,7-tetrafluoro-L-Trp (4,5,6,7tetraFW) were synthesized by coupling the fluorinated indole to L-serine following a procedure as described elsewhere.[4] Final purification was performed using reverse phase high performance liquid chromatography (RP-HPLC). Thus, the product eluted from a PD 10 column was concentrated 10-20 times[4] and loaded on a C18 column (Vydac 218TP150, 300 Å, 5 µm, 10 mm i.d. × 250 mm). Solvents for RP-HPLC were A (100% acetonitrile) and B (0.1% TFA in water). The column was washed and equilibrated using 85% of B in A. The concentration of B was linearly decreased from 85% to 80% in 10 min, followed by a 5 min gradient from 80% to 60%. Subsequently, the concentration of B was returned in one step to 85% and kept at this level for 7 min at a flow rate of 4 ml/min. Trp and Trp analogs were detected using the absorption at 280 nm and fluorescence at 300 nm (excitation wavelength at 280 nm). The collected fractions were pooled and concentrated by freeze-drying.

Bacterial strains and culture conditions

The bacterial strains used in this study are listed in Table S2. *Lactococcus lactis* NZ9000 and its derivatives were cultivated in M17 medium (Difco, Detroit, MI, USA) containing 0.5% (wt/vol) glucose (GM17) at 30°C. Erythromycin (Sigma-Aldrich, Santa Clara, CA, USA) was added at a final concentration of 5 µg/ml when required. Chemically defined SA medium with 0.5% (wt/vol) glucose and 20 µg/ml 5-fluoroorotic acid (5-FOA) (Sigma-Aldrich, Santa Clara, CA, USA) as a sole pyrimidine source was used for the generation of chromosomal knockouts, as described previously.[5] *Escherichia coli* DH5 was used for cloning purposes; it was cultivated aerobically at 37°C in LB medium (catalog number LMM01; Formedium, Norfolk, UK) with erythromycin at a final concentration of 200 µg/ml when required.

Table S2. Strains used in this study

Strain	Species	Description
PA1002	*L. lactis*	PA1001 knockout *trpAB* [6]
NZ9000	*L. lactis*	MG1363 *pepN::nisRK* [7]
NZ9000^{-trp}		NZ9000 knockout *trpAB* (this work)

Recombinant DNA techniques and oligonucleotides

Chromosomal DNA from *L. lactis* was isolated using the GenElute genomic DNA kit (Sigma-Aldrich). Plasmids and PCR products were isolated and purified using the High Pure plasmid isolation and PCR purification kit (Roche Applied Science, Mannheim, Germany) and the NucleoSpin gel and PCR cleanup kit (Macherey-Nagel, Düren, Germany) according to the manufacturers instructions. PCRs were performed with Phusion or DreamTaq polymerase (both from Fermentas, St. Leon Roth, Germany) according to the manufacturer’s protocol. The obtained PCR fragments were mixed and treated with the Quick-Fusion enzyme mixture (BIO-Connect Services BV, Huissen, the Netherlands), yielding 15-nucleotide overhangs annealing to complementary overhangs. No ligation was required; Quick-Fusion-treated mixtures were directly used to transform *E. coli*. Oligonucleotides employed in this study are listed in Table S3 and were purchased from Biolegio BV (Nijmegen, the Netherlands). Competent E. coli cells were transformed using heat shock[8], while electrocompetent L. lactis cells were transformed using electroporation[9] with a Bio-Rad gene pulser (Bio-Rad Laboratories, Richmond, CA, US). All nucleotide sequencing was performed at Macrogen Europe (Amsterdam, the Netherlands).

Table S3. Oligonucleotides employed in this study

**Primer Name**	**Sequence (5’ - 3‘)**
pCS1966_1FW	GTGCCTAATGAGTGAGCTAACTC
pCS1966_1RV	GTGGAATTGTGAGCGGATAAC
79-KOtrpA&B_Up_F	CGCTCACAATTCCACCTTTCCTAATGCTTTTGG
80-KOtrpA&B_Up_R	TACCATTTGTAAAGTGGACTTGATTGTAGGTCATGTATTT C
81-KOtrpA&B_Down_F	GAAATACATGACCTACAATCAAGTCCACTTTACAAATGGT A
82-KOtrpA&B_Down_R	TCACTCATTAGGCACTCGCCTGTGACACCTTAG

Construction of integration plasmid for *trpAB* knockout

Linearized vector pCS1966[5] was amplified using primers pCS1966_1FW/pCS1966_1RV. Primer pairs 79-KOtrpA&B_Up_F/80-KOtrpA&B_Up_R and 81-KOtrpA&B_Down_F/82-KOtrpA&B_Down_R were used, respectively, to obtain upstream (UP_F) and downstream (DOWN_F) regions of *trpAB*. Primer pair 79-KOtrpA&B_Up_F/82-KOtrpA&B_Down_R was used to perform an overlap PCR to obtain the flanking region UPDOWN_F. Primers KOtrpA&B_Up_F and 82-KOtrpA&B_Down_R contain 15 nucleotides at one end, overlapping with the sequence on the 5'-end of the linearized vector, followed by the flanking region of *trpAB* gene cassette and 15 nucleotides overlapping with the sequence on the 3'-end of the linearized vector. The fragment UPDOWN_F was fused with the linearized vector using Quick-Fusion, after which the reaction mixture was directly used to transform competent *E. coli* DH5. The resulting vector was designated pΔtrpAB.

Construction of *L. lactis* trpAB knockout mutant NZ9000^{-trp}

Integration plasmid pΔtrpAB, the *trpAB* knockout plasmid, was introduced in NZ9000 via electroporation. Knockout mutants were obtained by a two-step homologous recombination strategy.[5] First, plasmid chromosomal integrates were selected on erythromycin containing GM17 plates. Subsequently, the marker-free knockout strain was obtained through counterselection on 5-FOA on SA medium plates. Relevant chromosomal region was confirmed by nucleotide sequencing.

Molecular biology techniques. Construction of a plasmid encoding the gene for LmrR with a His_6-tag at the C-terminus was achieved by deletion of the strep-tag and the second glycine residue in plasmid pSCNZ8048-LmrR-strep[10], and addition of a His_6-tag at the C-terminus of LmrR by designing primers containing the modifications and performing a round of PCR as described previously.[11] The PCR products were purified with a kit (Roche Switzerland) and ligated with T4 ligase (Thermo Scientific). The His_6-tagged LmrR gene was inserted between the NcoI/XbaI restriction sites in plasmid pNSC8048.

L. lactis Trp auxotroph strain PA1002[6] was used for the expression of LmrR and the plasmid was electrotransformed into PA1002 containing the pMG36e-trpRS plasmid[4] using a Bio-Rad Gene Pulser. Because overexpression levels of RibU were modest when using PA1002[12], *L. lactis* Trp auxotroph strain NZ9000^{-trp} was used instead. *L. lactis* Trp auxotroph strain NZ9000^{-trp} was transformed using a Bio- Rad Gene Pulser with plasmid pMG36e-trpRS[6] and plasmid pNZ8048 containing the RibU W97Y-His10 gene.[13] The latter plasmid was kindly provided by dr D.J. Slotboom.

Protein production and purification.

LmrR. A 1 ml culture of *L. lactis* Trp auxotroph PA1002, transformed with pMG36e-trpRS and pNSC8048 LmrR-His_6 plasmids, was grown overnight without shaking at 30 °C in GM17 (M17 medium supplemented with 0.5% (w/v) glucose), with 5 µg/ml chloramphenicol and 75 µg/ml erythromycin. The overnight culture was inoculated into 50 ml fresh GM17 with 5 µg/ml of chloramphenicol and 75 µg/ml erythromycin, and incubated at 30 °C until an OD_{600} of 0.8 was reached. The cells were centrifuged at 5500×g for 8 min, and the pellet was resuspended in PBS buffer. This procedure was repeated two more times. Subsequently, the cells were resuspended in mCDM13[14], without Trp, and this culture was left for 30 min at 30 °C. Trp or Trp analog (1 mM) was added to the culture, followed by a 3.5 h period of starvation. The expression of recombinant LmrR protein was induced by adding 8 ng/ml nisin and the culture was left at 30 °C for 16 h.[4]

Cells were harvested by centrifugation at 5500 rpm for 10 min. The cell pellet was washed with approximately 250 ml of 20 mM Tris-HCl (pH 8.0), 50 mM NaCl, per 1 L culture of cells and stored at -80 °C. The cell pellet was resuspended in cell lysis buffer (20 mM Tris-HCl, pH 8.0, 500 mM NaCl, 25 mM imidazole) containing 10 mg/ml lysozyme (Sigma). The suspension of cells was incubated for 1 h at 30 °C, followed by addition of DNase I (Roche, 100 µg/ml), $MgSO_4$ (10 mM) and EDTA-free proteinase inhibitor (Roche, one tablet per 2 L culture of cells). Cells were broken by passing them three times through a French press at 16000 psi. Cell membranes and insoluble proteins were removed by centrifugation at 18,500 rpm for 40 min at 4 °C. The supernatant was loaded onto a Ni^{2+}-NTA column (1 ml bed volume) (GE Healthcare), equilibrated with 20 mM Tris-HCl, pH 8.0, 500 mM NaCl, and 25 mM imidazole. After washing with 10 column volumes of buffer (20 mM Tris-HCl, pH 8.0, 500 mM NaCl, and 50 mM imidazole), the LmrR protein was eluted using an imidazole concentration gradient from 50 to 500 mM. DNA bound to LmrR was removed by loading the LmrR-containing fractions on a heparin column (5 ml bed volume, GE Healthcare) as described elsewhere.[10] As final purification step, a gel-filtration column (Superdex 200 10/300 GL, GE Healthcare) equilibrated with 20 mM Tris-HCl, pH 8.0, 300 mM NaCl, was used. Highly purified LmrR fractions were pooled and concentrated to ~6 mg/ml using a Sartorius VIVASPIN TURBO 4 concentrator (10 kDa MWCO).

RibU. A 20 ml culture of *L. lactis* Trp auxotroph strain NZ9000^{-Trp} transformed with pMG36e-trpRS and pNZ8048 RibU W97Y-His10 plasmids in MG17 containing 0.5% glucose (w/v), 75 µg/ml erythromycin and 5 µg/ml chloramphenicol was grown overnight without shaking at 30 °C. The next day, 0.5 L of this medium was inoculated with 10 mL overnight culture and incubation at 30 °C was continued until an OD600 of ~0.6 was reached. Cells were harvested by centrifugation at 6500xg for 10 min at 30 °C and the pellet was resuspended in PBS buffer. This procedure was repeated two more times. The pellet was then resuspended in 0.5 L MCDM20 medium[14] supplemented with 75 µg/ml erythromycin, 5 µg/ml chloramphenicol, 1 mM Trp or Trp analogue and incubated for 1.5 h at 30 °C. Protein expression was induced by adding 5 ng/ml nisin and the culture was left overnight at 30 °C. Cells were harvested by centrifugation at 5500 rpm for 10 min. Membrane vesicle preparation and RibU isolation at 4 °C was performed as described before[13] with the following modifications: 5 mM imidazole was used during incubation of the supernatant with Ni-NTA resin and 40 mM imidazole was used during washing the Ni-NTA column. For gel filtration a PD MidiTrap G-25 desalting column (GE Healthcare) was used equilibrated with 20mM Tris-HCl pH=7.5, 150mM NaCl and 0.05% DDM.

MALDI-TOF. A purified LmrR solution (~6 mg/ml) was diluted to ~0.5 mg/ml with 100 mM NH_4HCO_3, and incubated with 5 µg/ml of trypsin during 2 h at 37 °C. 1 µl of digested LmrR was spotted, dried and quickly washed with 1 µl MilliQ water twice. Subsequently, 1 µl of matrix solution (10 mg/ml α-cyano-4-hydroxycinnamate (LaserBio Labs) in 50 % acetonitrile/0.1 % (v/v) trifluoroacetic acid) was spotted on top of the washed sample. Spots were measured using a Voyager DE-PRO MALDI-TOF (time of flight) instrument (Applied Biosystems). The incorporation efficiency of Trp analog was calculated after measuring the peak areas of the peptide containing either Trp or Trp analog.

Binding assays - surface plasmon resonance (SPR). LmrR and mutants were immobilized on a CM5 sensor chip and binding of drugs was monitored using a BIACORE3000 system. The binding responses of Dau and RBF to LmrR were measured in triplicate at both 25 °C and 10 °C, in HBS-EP running buffer (10 mM HEPES, pH 7.4, 150 mM NaCl, 50 µM EDTA, and 0.005% surfactant P-20). The binding responses were corrected by subtracting the response measured in the control lane, to which a comparable amount of a non-binding protein was immobilized. This protein, 4-oxalocrotonate tautomerase mutant Ala33Asp[15] was a gift of J.-Y. van der Meer. Steady state responses after injection of serial dilutions of Dau and RBF were used for the determination of the dissociation constant (K_d) values, using the BIAevaluation v.4.1 (GE Healthcare) and GraphPad Prism v5.00 software. The One site-Total equation from GraphPad Prism software was used for the calculation of Kd as follows: Y=Bmax*X/(Kd+X) + NS*X + Background. Equilibrium response Y is obtained at ligand concentration X. Bmax represents the maximum specific binding response to the same sensorchip. NS is the slope of nonspecific binding in Y units divided by X units. Background shows the amount of nonspecific binding without addition of ligand. Binding free energies, ΔGs, were derived from the dissociation constant K_d according to $\Delta G = -RT \ln K_d$.

Where possible, rate constants were determined using the BIAevaluation v.4.1 (GE Healthcare) software. k_{off} values were determined by fitting a 1:1 (Langmuir) binding model. Each k_{off} value is the average of 3 determinations.

Binding assays – Fluorescence titrations of RibU with RBF.

The concentration of the riboflavin stock was established using a Jasco V-650 spectrophotometer with an ε^{435} of 12600 $M^{-1}.cm^{-1}$ The concentration of RibU-His was calculated using a Nanodrop (Thermo Scientific) with an ε^{280} of 1.14 $ml.mg^{-1}.cm^{-1}$.[13]

Riboflavin was excited at 435 nm and emission was recorded between 450 nm and 700 nm using a FluoroLog 322 Spectrofluorometer (Jobin Yvon). The excitation and emission slits were set at 1.1 nm and 3.5 nm, respectively. Titration experiments were performed in a 1 mL quartz cuvette at 20 °C as described.[13] In brief, purified and desalted RibU protein in gel filtration buffer was titrated with RBF and after each addition the emission spectrum was recorded twice, corrected for buffer emission and instrument response and integrated. The integrated emission intensities were corrected for dilution and compared to the integrated emission of riboflavin collected under these conditions in the absence of RibU.

The K_d of RBF was derived by first determining the difference in fluorescence signal between the blank titration with only the gel filtration buffer and the titration using the protein RibU-His in buffer. The obtained curve was fitted using equation I and the program OriginLab[16] with n = number of binding sites, X = total RibU concentration, and S = maximal fluorescence signal in A.U.

Equation I:

$$[RibU * RBF] = \frac{\left(n + \mathrm{K_d} + \mathrm{x} - \sqrt{((\mathrm{K_d} + x + n)^2 - 4 \times n \times x)}\right)}{(2 \times n) \times (S)}$$

Aggregation tests. A solution of purified LmrR (~6 mg/ml) was buffer-exchanged into 10 mM sodium phosphate buffer, pH 6.8, 100 mM NaCl[3], and was diluted to ~1 mg/ml. The stock solutions of Dau and RBF were around 10 mM and 1 mM in water, respectively. LmrR and Dau or RBF were mixed in a 1:10 molar ratio and the mixture was kept for 30 min at room temperature. Dynamic light scatting (DLS) experiments were carried out at 25 °C with apo LmrR or LmrR-ligand mixture, using a DynaPro MSTC-800 instrument and Dynamics software (Wyatt Instruments, Santa Barbara, USA).

Gel filtration experiments were carried out at 10 °C with apo LmrR or LmrR-ligand mixtures in a molar ratio from 1:0 to 1:3.5, using a Superdex 200 increase 3.2/300 column (GE Healthcare), equilibrated with buffer (10 mM sodium phosphate, pH 6.8, and 100 mM NaCl), and an ÄKTA Explorer system. The protein-ligand mixtures were prepared with a 30 min incubation time, followed by centrifugation at 13,400 × g for 10 min at 4 °C.

Crystallization of LmrR and determination of crystal structures. Crystallization was carried out by the sitting-drop vapor diffusion method at 20 °C. Initial crystals of apo protein and complexes between LmrR mutants and Dau were obtained using the PACT premier screen from Molecular Dimensions. LmrR and Dau were mixed in a molar ratio of 1:5, using a protein concentration of ~6 mg/ml in gel filtration buffer (20 mM Tris-HCl, pH8.0, 300 mM NaCl), and the mixture was kept on ice for ~1 h, followed by centrifugation at 13,400 × g for 30 min before setting up a sitting drop. Each sitting drop was a mixture of 1 µl protein (complex) and 1 µl of reservoir solution. For 5FW-LmrR-Dau and apo 5FW-LmrR, the reservoir solution contained 100 mM Hepes (pH 7.0), 200 mM NH_4Cl, 20% PEG 6000. The crystals of apo 5,6diFW-LmrR, 5,6diFW-LmrR-Dau and 5,6,7triFW-LmrR-Dau were grown in sitting drops with a reservoir solution consisting of 25% PEG 1500, 100 mM SPG buffer

(succinic acid, sodium dihydrogen phosphate, and glycine in molar ratio 2:7:7) at pH 6.1.

Crystals appeared overnight and were flash cooled in a cryoprotectant solution consisting of the reservoir solution supplemented with 20% (v/v) glycerol. X-ray diffraction data were collected at the European Synchrotron Radiation Facility (ESRF) beam-lines ID23-1 and ID29, and at the Petra-III beamline P13 of the EMBL-Outstation at the Deutsches Elektronen-Synchrotron (DESY). Reflections were indexed and integrated using XDS[17], and the merging of the data was done with Aimless[18] from the CCP4 software suite[19] (see Table S1 for a summary of the crystallographic statistics). Initial models were obtained by molecular replacement using Phaser[20], with a single subunit of the dimeric LmrR apo structure (PDB entry 3F8B) as a search model. The models were further improved by several cycles of manual model-building using Coot[21] alternated with coordinate and temperature factor refinement using Phenix_refine.[22] Dau was fitted in 2Fo-Fc and Fo-Fc electron density maps and the LmrR-Dau complex structures were refined using optimized topology restraints for the ligand adapted from the CCP4 data library. Water molecules were added during the last cycles of model building and refinement. TLS refinement was included for a few of the models, where it led to a small decrease in the R-free R. All final models were validated using the Molprobity server.[23]

References

1. Madoori, P. K.; Agustiandari, H.; Driessen, A. J. M.; Thunnissen, A. M. W. H., Structure of the transcriptional regulator LmrR and its mechanism of multidrug recognition. *Embo J* **2009**, *28* (2), 156-166.
2. Zhong, W. G.; Gallivan, J. P.; Zhang, Y. O.; Li, L. T.; Lester, H. A.; Dougherty, D. A., From ab initio quantum mechanics to molecular neurobiology: A cation-pi binding site in the nicotinic receptor. *Proc Natl Acad Sci USA* **1998**, *95* (21), 12088-12093.
3. Takeuchi, K.; Tokunaga, Y.; Imai, M.; Takahashi, H.; Shimada, I., Dynamic multidrug recognition by multidrug transcriptional repressor LmrR. *Sci Reps* **2014**, *4*, 6922.
4. Petrovic, D. M.; Leenhouts, K.; van Roosmalen, M. L.; Broos, J., An expression system for the efficient incorporation of an expanded set of tryptophan analogues. *Amino Acids* **2013**, *44* (5), 1329-1336.
5. Solem, C.; Defoor, E.; Jensen, P. R.; Martinussen, J., Plasmid pCS1966, a new selection/ counterselection tool for lactic acid bacterium strain construction based on the oroP gene, encoding an orotate transporter from Lactococcus lactis. *Appl Environ Microbiol* **2008**, *74* (15), 4772-4775.
6. El Khattabi, M.; van Roosmalen, M. L.; Jager, D.; Metselaar, H.; Permentier, H.; Leenhouts, K.; Broos, J., Lactococcus lactis as expression host for the biosynthetic incorporation of tryptophan analogues into recombinant proteins. *Biochem J* **2008**, *409*, 193-198.
7. Kuipers, O. P.; de Ruyter, P. G. G. A.; Kleerebezem, M.; de Vos, W. M., Quorum sensing-controlled gene expression in lactic acid bacteria. *J of Biotechnol* **1998**, *64* (1), 15-21.
8. Van Die, I. M.; Bergmans, H. E. N.; Hoekstra, W. P. M., Transformation In Escherichia coli: studies on the role of the heat shock in induction of competence. *Microbiology* **1983**, *129* (3), 663-670.
9. Holo, H.; Nes, I. F., High-frequency transformation, by electroporation, of Lactococcus-lactis subsp Cremoris grown with glycine in osmotically stabilized media. *Appl and Environ Microbiol.* **1989**, *55* (12), 3119-3123.
10. Madoori, P. K.; Agustiandari, H.; Driessen, A. J. M.; Thunnissen, A., Structure of the transcriptional regulator LmrR and its mechanism of multidrug recognition. *EMBO J* **2009**, *28* (2), 156-166.

11. Zhou, L.; van Heel, A. J.; Kuipers, O. P., The length of a lantibiotic hinge region has profound influence on antimicrobial activity and host specificity. *Front Microbiol* **2015**, *6*, 11.
12. We attribute the low membrane protein expression by PA1002 to the deletion of the acmA gene in PA1002. Gene acmA encodes for the cell wall hydrolase N-acetylglucosaminidase and its deletion causes cell chaining. Buist, G.; Kok, J.; Leenhouts, K. J.; Dabrowska, M.; Venema, G.; Haandrikman, A. J., Molecular-cloning and nucleotide-sequence of the gene encoding the major peptidoglycan hydrolase of Lactococcus-lactis, a muramidase needed for cell-separation. *J Bacteriol* **1995**, 177 (6), 1554-1563.
13. Duurkens, R. H.; Tol, M. B.; Geertsma, E. R.; Permentier, H. P.; Slotboom, D. J., Flavin binding to the high affinity riboflavin transporter RibU. *J Biol Chem* **2007**, *282* (14), 10380-10386.
14. Shao, J.; Marcondes, M. F.; Oliveira, V.; Broos, J., Development of chemically defined media to express Trp-analog-labeled proteins in a Lactococcus lactis Trp auxotroph. *J Mol Microbiol Biotechnol* **2016**, *26* (4), 269-276.
15. Rahimi, M.; van der Meer, J. Y.; Geertsema, E. M.; Poddar, H.; Baas, B. J.; Poelarends, G. J., Mutations closer to the active site improve the promiscuous aldolase activity of 4-oxalocrotonate tautomerase more effectively than distant mutations. *Chembiochem* **2016**, *17* (13), 1225-1228.
16. Veldhuis, G.; Vos, E. P. P.; Broos, J.; Poolman, B.; Scheek, R. M., Evaluation of the flow-dialysis technique for analysis of protein-ligand interactions: An experimental and a Monte Carlo study. *Biophys J* **2004**, *86* (4), 1959-1968.
17. Kabsch, W., Xds. *Acta Crystallogr D* **2010**, *66*, 125-132.
18. Evans, P. R.; Murshudov, G. N., How good are my data and what is the resolution? *Acta Crystallogr D* **2013**, *69*, 1204-1214.
19. Winn, M. D.; Ballard, C. C.; Cowtan, K. D.; Dodson, E. J.; Emsley, P.; Evans, P. R.; Keegan, R. M.; Krissinel, E. B.; Leslie, A. G. W.; McCoy, A.; McNicholas, S. J.; Murshudov, G. N.; Pannu, N. S.; Potterton, E. A.; Powell, H. R.; Read, R. J.; Vagin, A.; Wilson, K. S., Overview of the CCP4 suite and current developments. *Acta Crystallogr D* **2011**, *67*, 235-242.
20. McCoy, A. J.; Grosse-Kunstleve, R. W.; Adams, P. D.; Winn, M. D.; Storoni, L. C.; Read, R. J., Phaser crystallographic software. *J Appl Crystallogr* **2007**, *40*, 658-674.
21. Emsley, P.; Lohkamp, B.; Scott, W. G.; Cowtan, K., Features and development of Coot. *Acta Crystallogr D* **2010**, *66*, 486-501.
22. Afonine, P. V.; Grosse-Kunstleve, R. W.; Echols, N.; Headd, J. J.; Moriarty, N. W.; Mustyakimov, M.; Terwilliger, T. C.; Urzhumtsev, A.; Zwart, P. H.; Adams, P. D., Towards automated crystallographic structure refinement with phenix.refine. *Acta Crystallogr D* **2012**, *68*, 352-367.
23. Williams, C. J.; Headd, J. J.; Moriarty, N. W.; Prisant, M. G.; Videau, L. L.; Deis, L. N.; Verma, V.; Keedy, D. A.; Hintze, B. J.; Chen, V. B.; Jain, S.; Lewis, S. M.; Arendall III, W. B.; Snoeyink, J.; Adams, P. D.; Lovell, S. C.; Richardson, J. S.; Richardson, D. C., MolProbity: More and better reference data for improved all-atom structure validation. *Prot Sci* **2018**, *27* (1), 293-315.
